# Assessing human–nature connection: A systematic review and a new Wetland Wanderer Tool for auditing nature connection in wetland environments

**DOI:** 10.1007/s13280-025-02335-1

**Published:** 2026-02-20

**Authors:** Kate Pratt, Vishnu Prahalad

**Affiliations:** School of Geography, Planning and Spatial Science, University of Tasmania, Private Bag 78, Hobart, TAS 7001 Australia

**Keywords:** Auditing tool, Human–nature connection, Inventory, Literature review, Wetland conservation

## Abstract

Wetlands are critical ecosystems that sustain biodiversity and support human wellbeing, yet limited public awareness of their values constrains conservation and restoration efforts. Drawing on human–nature connection (HNC) theory, this study develops a systematic approach for auditing and strengthening HNC in wetland environments through a new Wetland Wanderer Tool (WWT). The research comprised three stages: a systematic literature review identifying existing tools for assessing nature connection; development of the WWT through iterative expert consultation and field testing; and application of the tool across 21 Tasmanian wetlands as a proof of concept. Field application of the tool produced the first systematic inventory of HNC opportunities in Australian wetlands, revealing consistent shortfalls in orientation and only moderate provision of access. These findings highlight key engagement and infrastructure gaps, while also identifying examples of best practice. The WWT offers a scalable and transferable framework for integrating HNC into wetland planning and conservation.

**Supplementary Information:**

The online version contains supplementary material available at 10.1007/s13280-025-02335-1.

## Introduction

Humans are dependent on wetland ecosystems for a variety of goods and services including food, drinking water, fibre, medicine, timber, ground water recharge, flood control, and climate change mitigation (Millennium Ecosystem Assessment 2005; Lamsal et al. 2017; Ramsar Convention on Wetlands 2018). Wetlands also support fauna and flora found nowhere else on Earth and are among the most biodiverse ecosystems on the planet. Despite their ecological and societal importance, destructive and unsustainable practices largely related to urbanisation and agriculture have seen a vast alteration of wetland environments worldwide (Finlayson and Rea 1999; Bino et al. 2016; Hu et al. 2017; Briggs et al. 2025). The need to conserve, restore, and protect wetland ecosystems is recognised globally such as under the Ramsar Convention on Wetlands 1971 (hereto referred to as Ramsar), which outline multiple strategies to promote the "wise use" and management of wetlands, improve conservation management of wetlands, and protect and restore wetlands (e.g. Prahalad and Kriwoken 2010). Despite these initiatives dating back several decades, the global status of wetlands continues to decline, with wetlands disappearing three times faster than forests (Tickner et al. 2020).

Over the past few decades, Ramsar's recommendations have consistently promoted nature-based experiences to raise public awareness and support for wetland conservation. A joint report by the UN World Tourism Organisation and Ramsar (2012, p. 13) advocated for tourism in and around wetlands as a means to "raise awareness about wetland values and biodiversity and gain support from tourists and others for wetland conservation". Similarly, the Ramsar Convention’s Communication, Capacity-building, Education, Participation and Awareness (CEPA) Programme emphasised tourism and recreation in its 2012 Resolution (X1.7), urging "Contracting Parties and relevant stakeholders… to use Ramsar Sites as a branding opportunity to promote sustainable tourism and recreation practices, aiming to enhance appreciation of wetlands by offering meaningful visitor experiences, such as birdwatching and cultural activities" (Ramsar Convention Secretariat 2012, p. 5). While countries like Australia have formally endorsed this approach (being the first to produce a Wetland CEPA National Action Plan in 2001), there is still limited understanding of how wetland areas facilitate meaningful visitor experiences and foster greater awareness of wetland values and threats (Clarke et al. 2021; Pratt and Prahalad 2025).

In recent decades, the theories and ideas presented in literature on human–nature connection (HNC) highlight a potential path for enhancing wetland conservation. The HNC framework provides insight into how people relate to and interact with nature and how our relationship and interactions with nature shape our responses, feelings, and attitudes towards nature (Nisbet and Zelenski 2014; Barragan-Jason et al. 2022). Positive HNC is linked to human health and wellbeing as well as pro-environmental/conservation behaviour (Richardson et al. 2020; Soga and Gaston 2020; Barragan-Jason et al. 2022). Within the literature on HNC, blue and green spaces are recognised as varying contexts for fostering nature connections, where *green space* refers to terrestrial environments such as trees, forests and parks, and *blue space* refers to aquatic environments such as wetlands, lakes, and rivers (Finlay et al. 2015; Gascon et al. 2018; Smith et al. 2021).

However, numerous studies report that HNC is diminishing over time, because people are having less and less contact with natural environments in general (Miller 2005; Stokes 2006; Samways 2007; Soga and Gaston 2016; Soga et al. 2016). This trend, termed the extinction of experience (EOE), has been found to discourage positive emotions, attitudes, and behaviours towards the environment, with strong evidence suggesting that EOE undermines support for pro-biodiversity and pro-conservation policy and management (Pyle 1993; Stokes 2006; Soga and Gaston 2016; Soga et al. 2016; Gaston et al. 2018; Colléony et al. 2020; Richardson et al. 2020; Salazar et al. 2021; Barragan-Jason et al. 2022). Therefore, by provisioning individuals with positive meaningful nature-based experiences which foster HNC and reduce EOE, public support and action for wetland conservation could increase (e.g. McKinley et al. 2024).

**Fig. 1:**
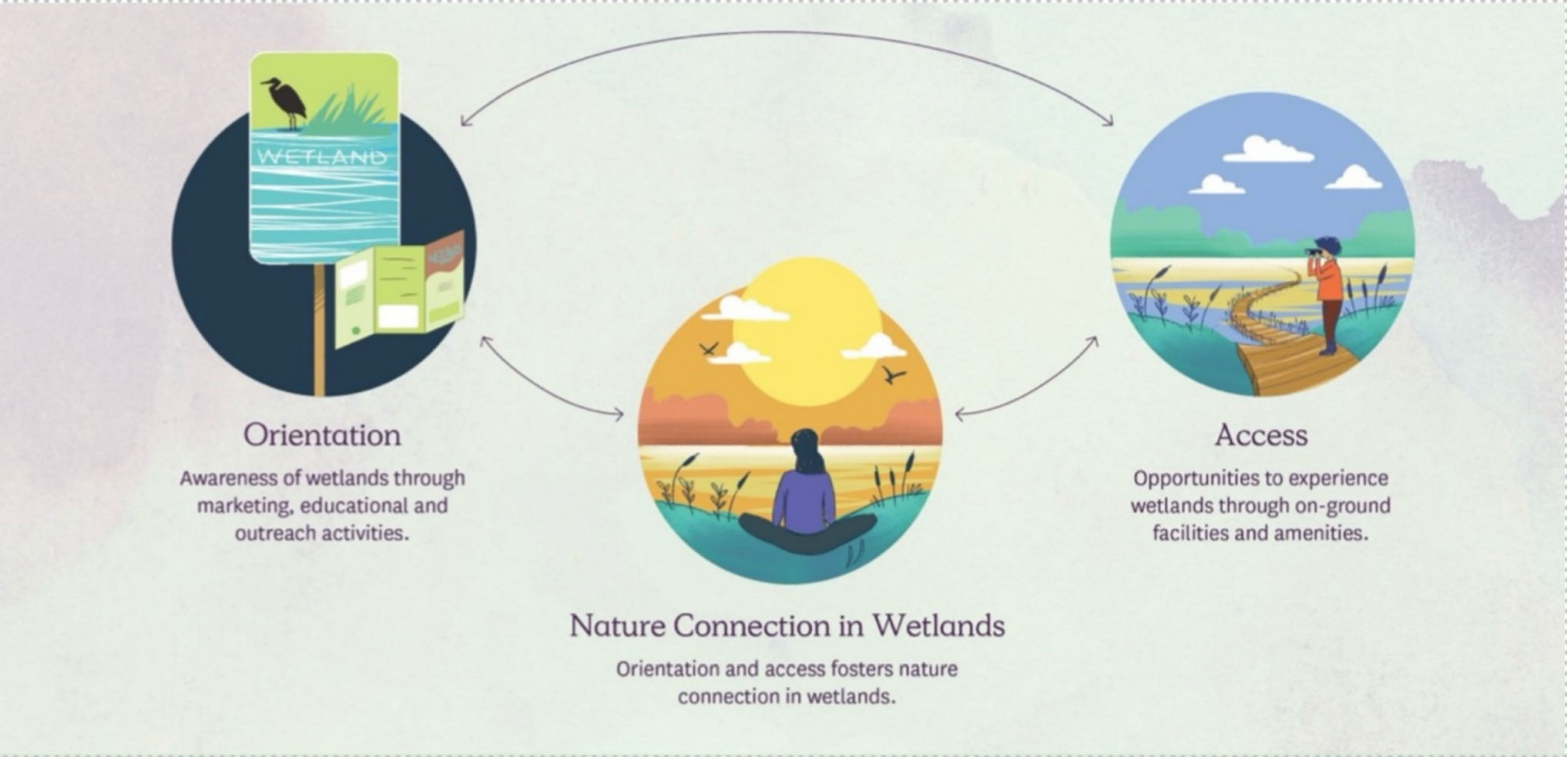
Conceptual diagram depicting theoretical relationship between orientation, access, and nature connection to wetlands, and how orientation and access can drive positive human–nature connections

To foster HNC, there needs to be an understanding of how people connect with and experience nature in wetlands (Wood et al. 2024; Pratt and Prahalad 2025). This can be achieved by studying how people are oriented towards wetlands as places for nature connection, and how people are able to access wetlands to have such experiences. *Orientation* towards and *access* to nature experiences and interactions have been noted as key drivers of positive HNC (Soga and Gaston 2016; Colléony et al. 2020). Loss of *access* is largely attributed to increasing urbanisation, and the modified nature of urban areas and urban living creating barriers for interacting with nature (Miller 2005; Stokes 2006; Samways 2007; Soga and Gaston 2016). Loss of *orientation* is associated with less frequent visitation to natural areas, with people who have limited awareness or familiarity with nature being less motivated to seek direct interactions with it (Soga and Gaston 2016; Colléony et al. 2020). *Orientation* towards nature is, however, primarily instigated by having *access* to nature to have positive experiences with nature (Nisbet et al. 2009; Kals 2014). Therefore, a key driver of orientation is the provision of opportunities (i.e. accessibility) for such experiences. As such, both *access* and *orientation* act upon one another to enhance HNC in a positive reinforcement cycle (Fig. 1).

We know from the literature that the provisioning of assets, inclusive of things like trails and boardwalks (access), hiking guides and trail maps (orientation) both enhance and enable positive nature experiences (Bell et al. 2018; Palliwoda and Priess 2021). Auditing of the assets thus becomes a key consideration towards understanding what experiences are available, and to what people they are available for. In fields such as urban design and protected areas management, auditing tools have been developed and employed and through their use, planners and policy makers can inventory spaces in terms of their affordances towards pre-defined purposes (Gidlow et al. 2018; Child et al. 2019).

Yet, there has been a lack of understanding of available tools that might assist in this endeavour towards auditing opportunities for the purpose of HNC. Although there is a plethora of tools which measure individual connection (i.e. Schultz 2001; Mayer and McPherson 2004; Martin and Czellar 2016; Salazar et al. 2020; Meis-Harris et al. 2021; Carr and Hughes 2021; ARI 2024) those which measure an environment's ability to support such connection seem absent. This is a considerable limitation towards achieving HNC (Gaston et al. 2018). Lack of knowledge of what opportunities are available for HNC both within a particular site or across a region, or state/country, prevents systematic planning towards provisioning of assets to facilitate meaningful engagement and connections with nature in general but more so in the often-overlooked wetland environments (Kingsford et al. 2016). This in turn further reinforces disaffection towards wetlands, leading to their ongoing loss (Finlayson and Rea 1999; Briggs et al. 2025).

The main aim of this study was to develop a systematic approach to auditing and strengthening HNC in wetland environments. To address this aim, the research was structured around three key objectives:

1. To conduct a systematic literature review to identify existing tools that could be used or adapted for assessing nature connection in wetlands.
2. To develop and test a new, fit-for-purpose auditing tool—the Wetland Wanderer Tool (WWT)—designed to evaluate HNC in wetland settings.
3. To demonstrate the application of WWT across Tasmanian wetlands, selected to represent a diverse range of ecological settings, management contexts, and visitor accessibility.

These objectives were operationalised through a mixed-methods research design comprising three stages that progressed from reviewing existing approaches, to developing the tool, and finally to demonstrating its application. This approach forms the structure of this paper, with each stage informing the next, in the development and application of an auditing tool to help inventory best practices and areas where HNC can be strengthened.

## Stage 1: Literature review

### Methods

We undertook a systematic literature review (SLR) using established methods (Pickering and Byrne 2013) to review auditing tools used to assess natural environments in which humans interact with nature. The review was carried out between August and October 2023. The databases Web of Science and Scopus were used to source academic literature which outlined the use or development of auditing tools used to assess human interaction with natural environments. Search terms included: "natural environment" or "park*" and "access" or "amenit*" and "tool" or "audit" or "instrument" or "inventory". Grey literature was sourced from consultation with experts (Table S4, Supplementary Material 1) and Google search engine using the same search terms. Database searches were limited to those articles which had search terms in the title, abstract, and keywords, and conference papers were excluded. All searches were limited to those published in the English language.

**Fig. 2:**
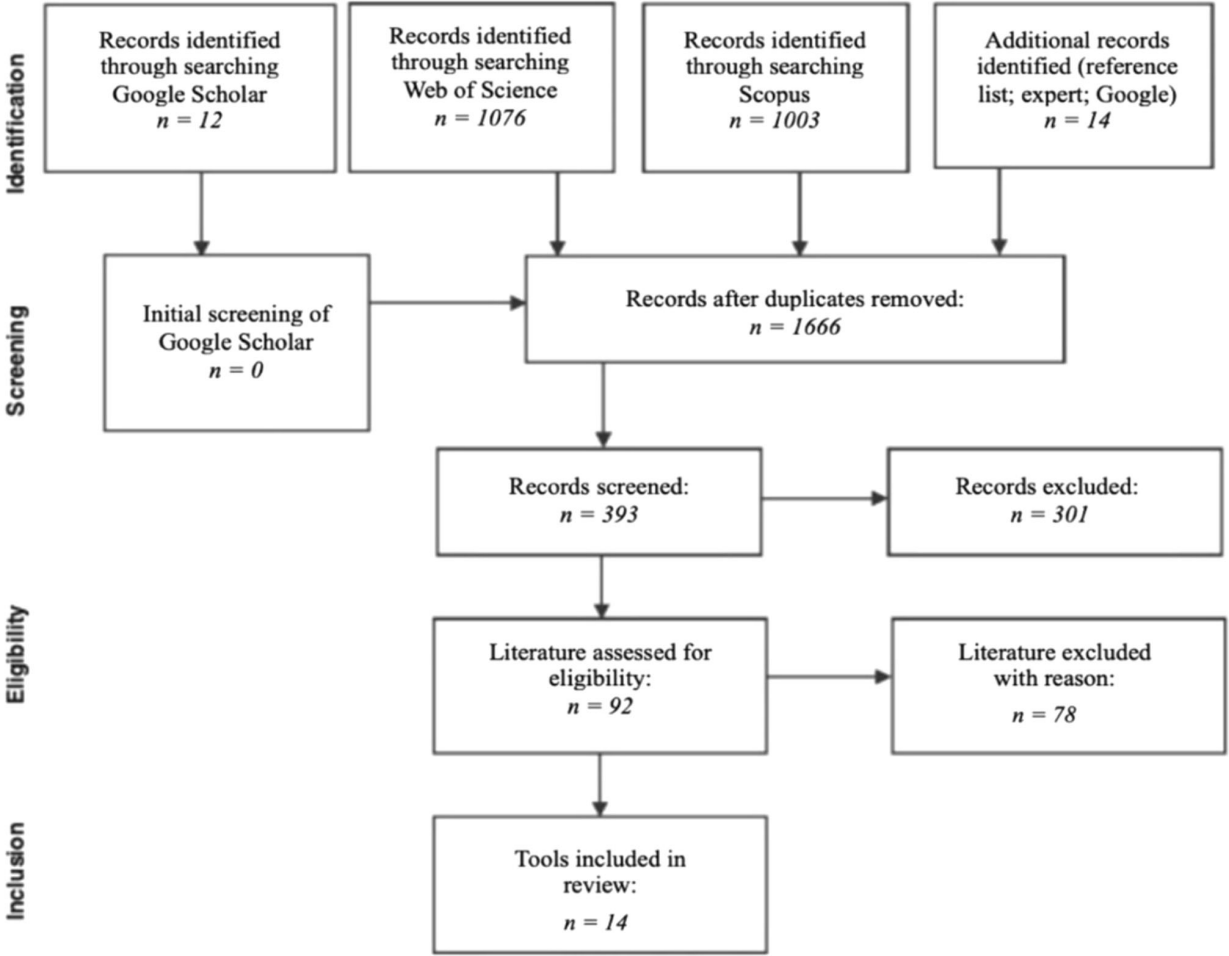
Customised PRISMA diagram depicting the review process of identification, screening, and assessment for eligibility and inclusion of papers (Moher et al. 2010)

The method of identification, screening, and assessment for eligible literature to be included in the review (Fig. 2) was adapted from the Preferred Reporting Items for Systematic Reviews and Meta-Analyses (PRISMA) (Moher et al. 2010). Database searching concluded when all articles had been screened for suitability (Web of Science and Scopus), or when six consecutive pages with 10 results per page resulted in no relevant results. We included both research articles outlining the development of a tool and stand-alone tools (those which were not referenced in academic literature but identified through consultation with experts: SAGE; Tables S3, S4, Supplementary Material 1). Tools which focused solely on accessibility in terms of reaching a destination were excluded. Tools auditing spatial characteristics alone, such as the number of parks in a city, were excluded. Tools were also excluded if their focus was limited to playgrounds and/or walkability. Lastly, the tool had to be available for use on-site and not reliant on desktop analysis alone. To ensure no tools were missed, reference lists from academic and grey literature (when applicable) were assessed for further tools that met inclusion criteria.

Tools eligible for inclusion in the review had the following information extracted: (1) authorship, year of development, country and hemisphere in which development occurred, tool name and the journal or other associated affiliation (grey literature); (2) the aim of the tool, the field of research it was developed for, the environment it is intended to be used in, and its intended users; (3) supporting material (guidance manual, training), development strategy, reliability/validity, problems in reliability, results/findings; (4) the format of the tool, the length of the tool and, scoring methods employed; (5) domains or sections and, items assessed. Information extracted from the tools for the review is available in Tables S1–S3 (Supplementary Material 1). Descriptive statistics were then used to organise data into meaningful results which have been detailed in the following section.

### Results

#### Publication time, journal, and location of development

**Fig. 3:**
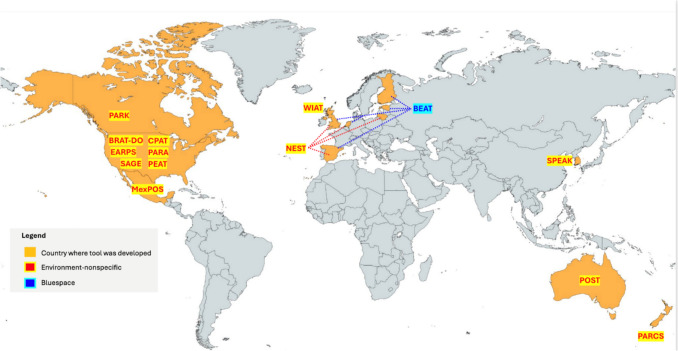
Location (i.e. country) of each tools development and intended environment for use (bluespace; environment nonspecific). Tool abbreviations are noted here, see text and Appendix 1 for more details on the tools

Through the review process, 14 tools were found appropriate for inclusion in this paper (Table A1.1 in Appendix 1). Their publication dates ranged from the years 2004 to 2022. Of the tools included, 11 have their development strategies published in academic journals. The most common journal was *The Journal of Physical Activity and Health* (21%; *n* = 3). Other journals largely related to public health, physical activity, or urban planning. Most tools were developed in the Northern Hemisphere (85%; *n* = 12), largely in the USA (43%; *n* = 6), or Europe (21%; *n* = 3) (Fig. 3). Few others were developed in Mexico (*n* = 1), Canada (*n* = 1, PARK), or Korea (*n* = 1, SPEAK). Tools developed in the Southern Hemisphere (14%; *n* = 2) were in Australia (*n* = 1, POST) and New Zealand (*n* = 1, PARCS) (Fig. 3).

#### Aim and field of research, intended environment for use, and intended users

The most common field of research relates to physical activity; the aim of tools concerning this field is to characterise or understand the environment and its attributes in terms of its use for and promotion of physical activity (64%; *n* = 9). Five tools also assessed the environment for its attribution to health and wellbeing benefits; however, no tool was based solely in the field of health and wellbeing; these have instead been categorised into either the field of physical activity or land use and planning.

Land use and planning were a field of research for 2 tools (NEST; SAGE); their aims focused on measuring the environmental quality of spaces or the current distribution of environmental assets to assist future planning measures. Another field of research concerns accessibility in terms of physical and mental disability, or physical impairment due to old age (PARCS: SPEAK). The final tool (WIAT) was developed for research concerning the change over time of woodlands which have undergone some form of intervention (i.e. regeneration), and how that relates to people’s quality of life.

The most common environment tools were developed for use was in urban areas (71%; *n* = 10). Three tools were developed for use in rural and urban areas (PEAT; POST; WIAT), and no tools were developed for use only in rural areas. One tool focused solely on blue spaces (BEAT), but its scope was too broad to suit the aims of this study. We categorised the rest (93%; *n* = 12) as environment nonspecific, meaning the environmental context (terrestrial/semi-aquatic; green/blue) was not a central consideration for the tool (Fig. 3). These tools typically focus on the human activity or outcome (e.g. for human health and wellbeing) rather than the characteristics of the environment in which these activities occur.

#### The format of the tool and scoring methods

Out of the tools included in this review, 79% (*n* = 11) are solely pen and paper based. The remaining use Microsoft Excel (NEST; also, PARK but has a pen and paper version), a free online access platform (BEAT), and Microsoft Access (PEAT). Tools varied from 751 items (EAPRS) to 18 (SAGE). Scoring methods were inclusive of presence/absence checks, 5-point Likert scale, multiple-choice checklists, binary and categorical responses, as well as comment sections for assessor observations. Most tools did not define a way to aggregate individual metrics into an overall measure. A few tools combined scores in to a final quantitative measure that can be used to differentiate between sites.

#### Themes and items assessed

Each tool contained sections or domains which categorised the items it assessed. We particularly focused on the aims of our study in our evaluation thus excluding items that were unrelated. For example, the quality of outdoor gym equipment and numerous other assessments of recreational facilities that are not nature based were excluded. Also excluded were items considering playground features and food elements considered from a health perspective. In total, 71 domains and 1646 items were included in our assessment and categorised into 14 themes (Table A1.2 in Appendix 1).

#### Development strategy and testing

Development strategies varied, with the most common approach involving consultation with experts in the relevant field of research as well as targeted community members (71%; *n* = 10). Consultation involved workshops, questionnaires, surveys, and/or forums aimed at developing inventories for each tool. Another common method used in development was the review of existing tools (50%; *n* = 7). This method is used to direct development strategies and find tools which can be adapted for the purposes of the tool being developed. Four tools used a combination of consultation and review.

Methods of testing tools also varied greatly. Testing ranged from 1 to 3 or more rounds; the number of testing sites varied immensely from 2 (BRAT-DO) to 944 (MexPOS). The testing of MexPOS also had the aim to generate a database of public open space in Mexico City, thus resulting in a large sample size. PARK, which also had many sites audited (576), states that this was to increase the reliability of the tool, arguing that other tools lack reliability due to the small number of sites tested during development. In comparison, tools with smaller numbers of testing sites like BRAT-DO (2) and PEAT (43) outline that selection was based on their sites representing all tool elements such that they can be reliably tested.

The number of assessors used to assess each site also varied greatly, ranging from 2 (EARPS) to 19 (NEST) individuals. The expertise of assessors was also variable, but dependant on the tools intended outcomes. For example, CPAT’s aim was to develop a tool for use by communities and employs community stakeholders as assessors. Most other tools used experts in the field of research relevant to the tool. BEAT employed best practice based off EARPS, PEAT, and other available reviews into tools. This method involves two expert assessors trained in BEAT independently assessing each site separately. The primary assessor (one of the two) acts as a baseline for all subsequent results. One commonality between tools was the importance of assessor training, stating that training greatly improves reliability.

Reliability testing, a method used to determine the consistency of testing results derived from different assessors, was used for all tools expect for SAGE and WIAT. Inter-rater reliability was tested by measuring either Kappa coefficients (binary questions), inter-class correlation coefficients (ordinal questions), or percentage agreement. In addition to reliability, some sought to test tool validity (e.g. BRAT-DO), to determine how accurate a tool is at measuring what is intends to measure. Many did not attempt to do this test as this relies on having a strong pool of test data which were often unavailable.

Reliability testing revealed that the most unreliable elements were those that were subjective or temporally variable, such as noise and lighting. These issues were dealt with by having the same group of assessors review each area on the same day of the week and at the same time. Another issue found was that tools were only tested in limited settings, such as parks in Mexico City (MexPOS); therefore, they would need to be adapted to suit other urban areas. Lastly, reliability was also found to increase with assessor training as mentioned above, and in one instance downscaling rating scales from 5 to 3 thereby reducing variability (EAPRS).

## Stage 2: Tool development

### Methods

Recognising the need to develop a new fit-for-purpose tool, we employed a development strategy by drawing from approaches identified in similar studies (reviewed above). This strategy involved: learning from the review of existing tools, consultation with experts in relevant fields, and initial deployment and testing at two pilot locations in southern Australia (near Adelaide, capital city of South Australia) prior to broader application in Tasmanian wetlands (Stage 3).

Our review of the 14 existing tools identified a common hierarchical structure, scaled from domains to items, which we adapted for WWT. The domains represented key themes which the tools used to structure similar items under, and our review helped adapt and incorporate these domains and items suited to the aims of this study. Through this development process, regular meetings were held with the research team under the Australian Government National Environmental Science Program (NESP) project IP1.02.01—Nature Connection, during which the lead author provided updates on tool development and received structured feedback from other team members. This collaborative process was further supported by feedback received from three academic colleagues within the same institution as the research team and through presentation at a national conference (Table S4, Supplementary Material 1).

Also, at the conference (in Adelaide, November 2023), a draft version of the tool was reviewed and tested by the authors and two other professional academics with expertise working in wetlands across Tasmania and Victoria. This testing involved using a paper copy of WWT along with instructions on how to complete the tool and to provide feedback on its usability and application. We used this approach as this kind of usability testing was common among other tools (e.g. Saelens et al. 2006; Kaczynski et al. 2012; Medina et al. 2022). Testing was done at Thompson Beach, Adelaide International Bird Sanctuary, part of Winaityinaityi Pangkara also known as the “Samphire Coast”, and at Coorong Lagoon near the Murray Mouth. Completed copies of the tool with handwritten notes were reviewed for observer agreement and feedback for improvements. Feedback for the tool provided key inputs for revision (Table 1).

**Table 1:** Examples of typical feedback received from testing the draft version of the Wetland Wanderer Tool (WWT) in Adelaide, Australia, and subsequent updates made

Version 1	Feedback	Version 2
What is the quality of the views?	“This question is very subjective”	Can you see the wetland from within the site? Is the view impeded by anything?
How extensive is the path/trail	“This is unclear”	Deleted
Is access impacting habitat protection?	“I think this needs to be more clear”	Are there signs indicating human-induced threats to the area? Are there fences/barriers protecting or restricting access? Are there signs indicating why these fences/barriers are in place?

### Results

The WWT comprised 76 items across 15 domains during testing in Adelaide (Table S5, Supplementary Material 1). Through an iterative process of tool development, this was revised to 124 items across 14 domains. Revisions were aimed at improving user clarity and reducing potential variation due to user subjectivity. The WWT was also hierarchically organised in two overarching sections, *orientation* and *access*, with the former having 3 domains and the latter 11 (Table 2). This sectioning reflects key dimensions of HNC considered in this study (Fig. 1).

**Table 2:** Structure of the Wetland Wanderer Tool (WWT) comprising of 2 sections (orientation and access) and 14 domains. Description of each domain is given below. *This domain was removed from the final version of the tool, see text for details. Complete final version of the Wetland Wanderer Tool is available as Supplementary Material 2

Section	Domain	Description
*Orientation*	Online Presence	Is the wetland visible in online search results? Assessors search for a wetland on their preferred search engine and try to locate webpages which promote recreation and volunteering at the wetland. This section also asks users to gather other information about the wetland such as cultural heritage status
	Education and Outreach	Are wetland values being promoted through tours, events, and visitor centres? Are people encouraged to take part in volunteer work and citizen science activities in wetland areas?
	Signage	What kind of signage is in the wetland? What is the condition of this signage?
*Opportunities*	Use of Site	Who is using the wetland and for what purpose? What activities take place at the wetland?
	Safety and Security	Does the wetland provide a safe and secure environment for visitors?
	Incivilities	Are visitors to the wetland likely to experience anti-social behaviour from other visitors? Is the wetland clean or is there rubbish?
	Aesthetics	Does the wetland enhance visitors’ opportunity to spend time and appreciate the wetland? Are there any unpleasant noises or smells?
	Access	Can visitors travel to the wetland via available transport options? When they get there, can they enter the wetland?
	Accessibility	Is anyone excluded from the site due to physical or cognitive requirements (walking ability, language barriers, vision)?
	Paths and Trails	Are there paths and trails within the wetland area? What is their condition?
	Facilities and Amenities	What facilities and amenities are available in the wetland? What is their condition?
	Maintenance and Management*	Is the wetland being managed? Who is the wetland being managed by?
	First Nations Heritage and Values	Is the First Nations Heritage and values within the site being acknowledged?
	Natural Values	Are the natural values within the wetland being protected or restored? Are people being educated on the flora and fauna values of wetlands?

## Stage 3: Tool application, validation, and refinement

### Methods

To apply, validate, and refine WWT, we used methods drawn from our literature review to select sites, undertake assessment, and test the tool’s reliability (i.e. between assessors) and capacity (i.e. in developing an inventory of opportunities for nature connection). This stage was designed as a proof of concept rather than an exhaustive inventory.

### Site selection

**Fig. 4:**
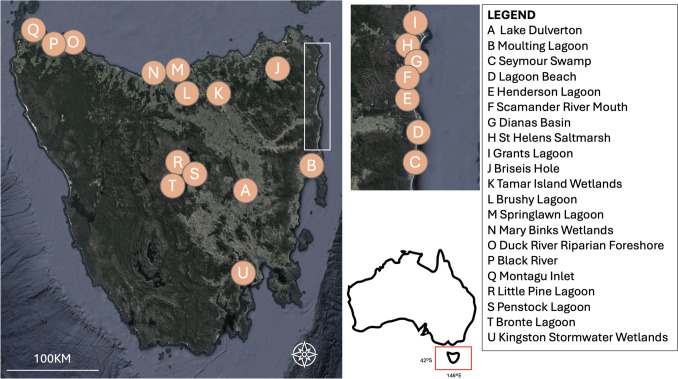
Location of the 21 wetland sites used for inter-rater reliability testing of WWT and inventorying of HNC across Tasmania, Australia. Source: Google Earth Pro (2013–15)

A total of 21 sites were selected across Tasmania, Australia, using a stratified, opportunistic sampling approach to ensure representation across different geographic regions, wetland types, and management contexts (Fig. 4). Selected sites covered 11 local government areas and a diversity of wetland types including inland wetlands, marine and coastal zone wetlands, and human-made wetlands (Table 3). Conscious effort was made to choose sites encompassing varying levels of remoteness, including 6 sites in remote areas, 13 in outer regional areas and 2 in an inner regional area (Australian Bureau of Statistics 2024). Among the 21 sites, all of them were on publicly accessible land, 11 sites were managed by Parks and Wildlife Services Tasmania, 3 sites managed by Hydro Tasmania (a government-owned business), one site managed by the Tasmanian Aboriginal Centre (TAC), and the remaining sites managed by local councils.

**Table 3:** Each of the 21 Tasmanian wetlands assessed using the Wetland Wanderer Tool (WWT) categorised by the type of wetland it is, its remoteness area classification, who the wetland is managed by, and what local government area it is in. *I—inland wetland; MC—marine and coastal wetland; H—human-made wetland (DCCEEW, 2019)

Wetland	Type of wetland*	Remoteness area	Managed by	Local government area
Lake Dulverton	I	Outer Regional	Southern Midlands Council	Southern Midlands
Moulting Lagoon	MC	Remote	Parks and Wildlife Services Tasmania	Glamorgan–Spring Bay
Seymour Swamp	MC	Remote	Parks and Wildlife Services Tasmania	Glamorgan–Spring Bay
Lagoon Beach	MC	Remote	Parks and Wildlife Services Tasmania	Break O’Day
Henderson Lagoon	MC	Outer Regional	Parks and Wildlife Services Tasmania	Break O’Day
Scamander River Mouth	MC	Outer Regional	Break O’Day Council	Break O’Day
Dianas Basin	MC	Outer Regional	Tasmanian Aboriginal Centre	Break O’Day
St Helens Saltmarsh	MC	Outer Regional	Parks and Wildlife Services Tasmania	Break O’Day
Grants Lagoon	MC	Outer Regional	Parks and Wildlife Services Tasmania	Break O’Day
Briseis Hole	H	Outer Regional	Dorset Council	Dorset
Tamar Island Wetlands	MC	Inner Regional	Parks and Wildlife Services Tasmania	West Tamar
Brushy Lagoon	H	Outer Regional	Parks and Wildlife Services Tasmania	Meander Valley
Springlawn Lagoon	MC	Outer Regional	Parks and Wildlife Services Tasmania	Latrobe
Mary Binks Wetlands	H	Outer Regional	Devonport City Council	Devonport
Duck River Riparian Foreshore	MC	Outer Regional	Circular Head Council	Circular Head
Black River	MC	Outer Regional	Parks and Wildlife Services Tasmania	Circular Head
Montagu Inlet	MC	Outer Regional	Parks and Wildlife Services Tasmania	Circular Head
Little Pine Lagoon	H	Remote	Hydro Tasmania	Central Highlands
Penstock Lagoon	H	Remote	Hydro Tasmania	Central Highlands
Bronte Lagoon	H	Remote	Hydro Tasmania	Central Highlands
Kingston Stormwater Wetlands	H	Inner Regional	Kingborough Council	Kingborough

### On-site assessment

The lead author and one trained volunteer visited the 21 wetlands in January 2024 to undertake independent assessments of each site, timed to coincide with peak visitation and capture representative visitor use. A pen and paper version of the tool was used for the assessment, where all the items and domains in WWT were scored in situ (Table S5, Supplementary Material 1). For each assessment, start time, weather conditions, and end time (duration of the effort) were noted by both assessors. The second assessor was necessary to check the assessment of the lead author, through an inter-rater reliability assessment (see the following section). These protocols are in line with other studies that have developed and tested similar tools (e.g. Lee et al. 2005; Bedimo-Rung et al. 2006; Bird et al. 2015).

To compare the results of the WWT for each domain and wetland visited, both assessors awarded points for each item (Table S6, Supplementary Material 1). The points awarded for items under each domain were added together and given a percentage score. These scores were then ranked into the following classifications: None (< / = 0%), Low (> 0% to 25%), Fair (> 25% to 50%), Moderate (> 50% to 75%) and, High (> 75%). These classifications were applied descriptively to enable comparison between sites and are not drawn from an established scale. Data of the scored assessments were then placed into a table that was colour-coded to illustrate a spectrum of opportunity, adapted from the recreation opportunity spectrum of Clark et al. (1979). The colours given to each classification are as follows: red (None), orange (Low), yellow (Fair), light green (Moderate) and dark green (High). Not every item in the WWT was given a score as some items are designed to give managers qualitative information that does not need to be quantified. For example, the domain "Aesthetics" includes the question: "what is the view impeded by"’, this is a prompt for the rater to note what is obstructing the view (e.g. overgrown vegetation) so that a flag is raised for potentially fixing the issue. Scoring items were therefore inclusive of 103 out of 124 items.

### Inter-rater reliability test

Inter-rater reliability between the two assessors was tested using percentage observer agreements and Cohen’s Kappa coefficients (k); both unweighted (for nominal items which included yes/no answers and categorical items) and weighted (for ordinal or ranked items such as Likert-data). These statistical methods are commonly employed for testing inter-rater reliability of tools (e.g. Bedimo-Rung et al. 2006; Troped et al. 2006). Cohen’s Kappa is highly relevant as a test metric as it accounts for chance agreement between observers (Bird et al. 2015; Li et al. 2023); however, it has also been noted to under-estimate agreements significantly (McHugh 2012; Li et al. 2023). Conversely, percentage agreement statistics have been criticised for over-estimating observer agreement. Given these trade-offs, both these test metrics have been included to evaluate inter-rater variances.

Percentage agreement was calculated by the number of times both assessors agreed, divided by the number of observations made. Kappa coefficients were assigned by calculating unweighted k-coefficients. The equation used is shown below (McHugh 2012):

$$k = \frac{\Pr \left( a \right) - \Pr \left( e \right)}{{1 - \Pr \left( e \right)}}$$

where Pr(a) equals the actual observed agreement, and Pr(e) represents chance agreement. Weighted Kappa coefficients were estimated using the equation shown below (Fleiss et al. 2003):

$$k_{w} = 1 - \frac{{\sum {w_{ij} \cdot fo_{ij} } }}{{\sum {w_{ij} \cdot fe_{ij} } }}$$

where *w* are the weighting factors, *fo* are the observed frequencies, and *fe* are the expected frequencies.

To interpret weighted and unweighted k-values, ranges described by Landis and Koch (1977) are used, modified slightly to account for perfect agreement: poor (< 0.00), slight (0.00–0.20), fair (0.21–0.40), moderate (0.41–0.60), substantial (0.61–0.80), near perfect (0.81–0.99) and perfect (1.00). To determine the sufficient level of inter-rater agreement, Graham et al. (2014) compared results from reliability testing literature, finding that anywhere above 75% percentage agreement was accepted as reliable and that a rating of 0.61 and above is an acceptable k-value. These ranges were used in our study to determine if results were reliable.

## Results

### Wetland inventory based on WWT

The 21 wetlands assessed varied widely in their scores across the WWT domains (Table 4). Out of the 5 levels (None, Low, Fair, Moderate, High) used to rate a site’s ability to support HNC, 33% (7) of the sites were scored as being Low (Bronte Lagoon, Brushy Lagoon, Black River, Dianas Basin, Scamander River Mouth, Lagoon Beach, and Moulting Lagoon), 55% (11) were scored as being Fair (Penstock Lagoon, Little Pine Lagoon, Briseis Hole, Mary Binks Wetlands, Kingston Stormwater Wetlands, Montagu Inlet, Duck River, Grants Lagoon, St Helens Saltmarsh, Henderson Lagoon, and Seymour Swamp), 10% (2) were scored as being Moderate (Lake Dulverton and Springlawn Lagoon) and 5% (1) was scored as being High (Tamar Island Wetlands). The lowest scoring wetland was Black River (15%), and the highest scoring wetland was Tamar Island (77%).

**Table 4:**
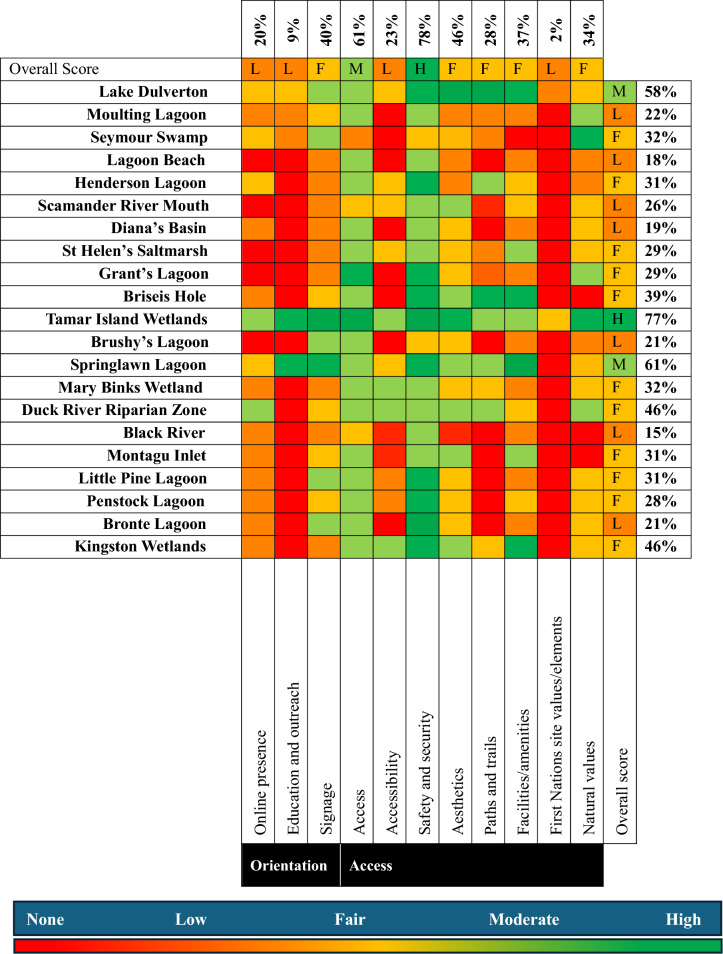
Spectrum of opportunities for human–nature connection in Tasmanian wetlands. Results are based off 21 sites across Tasmania, assessed via the Wetland Wanderer Tool (WWT). Three domains (Use of Site, Incivilities, Maintenance/Management) within access are not included here, see text for more details

Domains in the *orientation* section have an average score of 23% (Low), which was lesser compared to the *access* section with an average score of 39% (Fair). Domains within these sections had widely different ratings with Safety and Security assessed as being the highest (78%) and First Nations assessed as being the lowest (2%). The domain results were often affected by individual items and varied across sites (Table S7, Supplementary Material 1).

### Inter-rater reliability

Inter-rater reliability was assessed for 12 of 14 domains, which included 109 of 124 items for which sufficient data were available. A total of 15 items were excluded due to insufficient data, including the entire First Nations domain (4 items), 2 from Safety and Security, 1 from Incivilities, and 1 from Paths/Trails. Observation data were low for these items as the questions related to their assessment required the assessors to evaluate uncommon features within sites (i.e. presence of stairs, reference to traditional owners). As such, answers could not be given, and reliability could not be determined. Similarly, inter-rater reliability for the Online Presence domain (7 items) could not be determined, as only one assessor was available to complete this part of the assessment.

Table 5 shows the inter-rater reliability of each domain (for the results of each individual item see Table S8, Supplementary Material 1). Out of the 12 domains tested, 10 were found to have acceptable k-values (> / = 0.61), and 11 with acceptable percentage agreement (> / = 75). Use of Site was the only domain to have unacceptable scores in both percentage agreement and k-values (70%; 0.40). Incivilities had an unacceptable k-value (0.51) but an acceptable percentage agreement (80%). Maintenance and Management scored the highest with perfect agreement in both scoring methods. Use of Site scored the lowest out of each scoring method. Consequently, these three domains were excluded from our assessments (Table 4).

**Table 5:** Inter-rater reliability for nominal and ordinal items of Wetland Wanderer Tool (WWT), based on observations at 21 wetlands in Tasmania, Australia

Domain	# items	Weighted/Unweighted (W/UW)	Percentage agreement (%)	Cohen’s kappa (*k*)	Range (k)	Level of agreement
Online Presence	0 of 7	NA	NA	NA	NA	NA
Education and Outreach	10	UW	98	0.96	0.64–1.0	Near Perfect
Signage	12	UW	89	0.77	0.60–1	Substantial
Use of Site	5	W, UW	70	0.40	0.13–0.75	Fair
Access	12	W, UW	91	0.78	0.61–1	Substantial
Accessibility	8	W, UW	92	0.84	0.40–1	Near perfect
Safety and Security	7 of 9	W, UW	92	0.82	0.61–1	Near perfect
Incivilities	5 of 6	W, UW	80	0.51	0.005–1	Moderate
Aesthetics	16	UW	83	0.66	0.15–1	Substantial
Paths and Trails	9 of 10	UW	80	0.61	0.16–1	Substantial
Facilities and Amenities	16	UW	93	0.86	0.60–0.86	Near perfect
Maintenance and Management	1	UW	100	1	1	Perfect
First Nations Values	0 of 4	N/A	N/A	N/A	N/A	N/A
Natural Values	8	UW	84	0.62	0.23–0.90	Substantial

### Fine-tuning WWT

Following field testing and assessment of inter-rater reliability, several refinements were made to WWT. Some items were removed, others added, and a few were relocated between domains, resulting in a final structure of 110 items across 13 domains (Wetland Wanderer Tool in Supplementary Material 2). The domain Maintenance/Management was removed as it contained only 1 item that showed no variation across sites, making it unsuitable for comparison. Education and Outreach domain had 4 items removed as they were repetitive. Facilities/Amenities, Signage, and Access all had 1 item relocated; added to Accessibility. Aesthetics domain lost 3 items, as did paths/trails. Online Presence was updated to improve usability. Finally, the domain Use of Site was renamed to Site Popularity to better reflect what it measures.

## Discussion

This discussion situates the Wetland Wanderer Tool (WWT) within the broader context of human–nature connection (HNC) assessment frameworks, reflecting on its development, validation, and application across Tasmanian wetlands. We first position WWT in relation to existing tools assessing nature connection before discussing insights gained from its application in the field and their implications for wetland planning and management.

### Placing WWT in a world of tools

Stage 1 of this research aimed to review existing tools that could be used or adapted for assessing HNC in wetlands. A key finding from our review was that all tools, except for BEAT (Mishra et al. 2020), were developed as environment-nonspecific tools, meaning that the environmental setting (blue or green) was not a central consideration in their design. For example, tools concerned with outdoor physical activity do not discriminate or exclude spaces where physical activity could occur (i.e. kayaking in a *blue space*, golfing in a *green space*). Such tools are designed specifically with this feature so as not to bias results towards terrestrial or aquatic activities. Presently, as wetlands are by definition *blue spaces*, the environment becomes a key design consideration in developing a fit-for-purpose WWT.

Indeed, a key finding from our review was that there were no tools that are suitable for the application of auditing *blue spaces*/wetlands for assessing how public awareness (or, *orientation*) is currently being fostered, and what opportunities are currently available in these environments for nature-based experiences. Some of the tools that we reviewed did cover activities in *blue spaces* but were aimed to assess these spaces in terms of their usefulness for physical activity. Although physical activity in natural environments can itself be a nature-based experience (e.g. Rosa et al. 2019; Silva et al. 2023), HNC is not limited by individual proclivities and abilities for physical activities. Therefore, a tool which aims to assess an environment’s ability to foster HNC needs to consider multiple modes and forms of interaction (Ives et al. 2017; Pratt and Prahalad 2025). Also, interestingly, none of the tools we reviewed considered awareness or orientation as a key criterion in their design, with minor exceptions such as signage (e.g. Bedimo-Rung et al. 2006; Mishra et al. 2020). Given these limitations, along with the geographic bias towards northern-hemisphere studies (Fig. 2), Stage 1 of this research demonstrated the need for a context-specific, fit-for-purpose audit tool for assessing HNC in wetland environments.

In Stage 2 of our research, insights from the literature review, combined with expert consultation and field testing, guided the development of the new WWT, notably the first tool developed for the assessment of HNC in wetland environments. The decision to adopt a hierarchical structure, organised from domains to items, was consistent with most tools reviewed, yet its application here was refined to reflect the dual processes of orientation and access that underpin HNC (Fig. 1). This structure bridges theoretical concepts of awareness and engagement with practical assessment criteria that can be audited in the field. The collaborative and iterative development process helped ensure that the resulting tool is not only grounded in a rigorous scientific process but also practical and accessible for policy makers, wetland managers and other stakeholders as a semi-quantitative method of assessing HNC in wetlands.

The design of WWT is such that it can evaluate wetlands individually and at larger scales (e.g. regionally) in specific geographic and environmental contexts relevant to Tasmania. However, WWT is likely applicable across Australia and in comparable settings elsewhere, provided appropriate adaptations are made to reflect local biophysical and socio-cultural conditions. This recognises the role spatial dynamics and geographic variation play in greatly influencing human–nature interaction and experience (Soga and Gaston 2020). Also, while WWT was developed specifically for wetland environments (or, blue spaces), several of its features such as the hierarchical structure with the orientation access framework can be adapted for assessing HNC in other ecosystems (e.g. forests, alpine environments). Given the paucity of tools available for auditing and inventorying the environment's ability to foster HNC, WWT provides a model that can be adapted to diverse settings.

### Developing a wetland inventory for HNC

Stage 3 of our research applied and validated WWT as a proof of concept to evaluate its reliability and scalability (i.e. for inventorying) in real-world settings. The application of WWT in 21 wetlands across Tasmania has provided the first systematic inventory of opportunities for fostering HNC in wetlands (Table 4). The following discussion is structured into *orientation* and *access* sections of the tool and their respective domains to illustrate how they offer distinct yet complimentary insights into HNC.

#### Orientation

Firstly, our inventory indicates that there is a considerable and consistent lack of *orientation* (23% on average across the 3 domains assessed, with only three sites scoring fair or above: Table 4), signifying limited effectiveness in directing people towards wetlands as places for recreation, visitation, and learning.

#### Online presence

The low score (20% on average) recorded in the *online presence* domain indicates that potential visitors are not being effectively directed towards wetland areas when using the internet to plan trips and holidays in Tasmania. In many cases, this reflects the fact that several of the assessed wetlands have little or no dedicated online presence—such as official webpages, visitor information, or other content (e.g. reviews or blogs). Similarly, Prahalad and Kriwoken (2010) found that the majority of Ramsar listed wetlands in Tasmania received little or no recognition on webpages promoting nature-based tourism in Tasmania. Low scores in this domain are problematic, as digital marketing and search engine visibility are important aspects of tourism promotion (Konidaris and Koustoumpardi 2018). For instance, a study by Google (Think with Google 2015) found that 60% of leisure travellers use search engines to plan and research their travel. As this section of WWT requires the assessor to search for "information concerning recreational opportunities at the site", low scores in this domain indicate lack of digital context and/or visibility, therefore pushing wetlands into the digital periphery (Konidaris and Koustoumpardi 2018).

#### Education and outreach

We found that most wetlands (16 out of 21) do not provide visitors with opportunities to learn about and engage with wetlands via tours, volunteering, other events, and through visitor centres. The general absence of these opportunities and a low average score (9%) is likely to be contributing to low levels of public awareness and environmental literacy concerning wetlands. Indeed, there are numerous studies from around the world which find public knowledge about wetlands lacking (Finlayson and Rea 1999; Duarte et al. 2008; Boon et al. 2011; Ibrahim et al. 2012; Sharai et al. 2020). This pattern is also evident in Tasmania, where Wang et al. (2022) reported that many visitors lacked knowledge of and familiarity with wetlands, despite being in a region rich in these environments (also see Pratt and Prahalad 2025). Notably, those surveyed visitors who reported limited knowledge of wetlands expressed a desire for visitor or information centres, underscoring the importance of such facilities in expanding education and outreach across the broader population (Ramsar and UN World Tourism Organisation 2012).

#### Signage

Our results across the *signage* domain scored better than other domains under the *orientation* section (40% on average, and 13 sites scoring either fair or above). Low scores in this domain were due to wetlands lacking in either a certain type of signage (i.e. directional, interpretation or regulatory) or display signs which are damaged and obstructed thereby restricting usage. Signage can play an important role in teaching and engaging the public about wetlands (Alder 1996; Leisher et al. 2012; Martin et al. 2015). Also, signage gives a place meaning through providing it a name, directing people towards it, providing information about it, while also operating as a passive means of protection through outlining what behaviours are appropriate (Milstein 2011; Soica and Roland 2022). Signs, as Milstein (2011) states, act as a "pointing and naming" device for voiceless nature providing language via which humans can speak for it. We also know that people expect signs (or, information panels) to help direct them to visit certain areas, especially when they may not be aware of the available options (Weaver and Lawton 2011; Wang et al. 2022).

#### Access

Our inventory indicates that *access* was rated as fair across the 8 domains assessed (39% on average: Table 4), though with considerable variability between domains and individual items. These findings highlight key infrastructure, both hard (e.g. disability access, tracks and trails) and soft (e.g. safety), that could be improved or expanded to enhance HNC in wetlands.

#### Access, accessibility, and safety/security

Our results for the *access* domain (61% on average) indicate that almost all wetlands are accessible to the public in terms of allowing entry to sites and providing car parks. However, as public transport or cycleways were not available to most wetlands, access is largely restricted to people who have a motor vehicle. As low-income earners, particularly women and minority groups, may struggle to afford a motor vehicle, this finding highlights an inequality in access to wetlands (Hamilton and Jenkins 2000; Bostock 2001; Wang 2003). More generally, other studies have highlighted a relationship between low-income groups and lack of access to nature in Australia and elsewhere (Wolch et al. 2005; Jones et al. 2009; Astell-Burt et al. 2014; Rigolon and Flohr 2014). Thus, there is an important need to enhance *access* for a broader and more diverse range of people through the provision of public transport, cycleways, and other appropriate means of reaching wetlands.

In contrast with access, the accessibility domain scored poorly (23% on average) indicating many wetlands generally lacking in the provision of amenities (e.g. wheelchair access, braille signs) to facilitate people with disabilities to spend time in nature. This reflects results of Perry et al. (2018) who studied the accessibility of parks and playgrounds in New Zealand and found that none of the study areas met with national and/or international guidelines for accessibility design. *Australia’s Disability Strategy 2021–31* outlines in Priority 4 that universal design principles should be adopted in natural spaces so that everyone is enabled access regardless of ability (Department of Social Services 2021). We expect WWT to address this gap by explicitly evaluating site accessibility and, in doing so, flagging potential areas for infrastructure provision in line with the national strategy.

It is important for people to feel safe and secure if they are to develop positive connections with nature (Soga and Gaston 2022). Also, Dwyer et al. (2009) note that people’s perception of safety and security is fundamental to the success of tourism development, and importantly, people select destinations based on personal safety and security. In this context, our results for the *safety and security* domain, which indicated that the Tasmanian wetlands we audited were likely to provide a safe and secure environment for visitors (78% on average), augur well for enabling positive HNC. We do acknowledge that safety and security can potentially be subjective, and that both effective access (e.g. formed tracks, boardwalks) and information (e.g. signage, websites) is necessary to overcome any fear, both real and misconceived.

#### Aesthetics, paths/trails, and facilities/amenities

Our results indicate that there is variability among wetlands in terms of their *aesthetics* (with an average score of 46%) and with substantially high inter-rater reliability (Table 5). Aesthetics in relation to WWT does not pertain to perceptions of "beauty" but more so on how individual assessments of beauty (or not) are facilitated through thoughtful amenities that allow space and prompts for visitors to observe and reflect on the environmental qualities of wetlands. This follows the understanding in the literature that subjective feelings about the qualities of natural spaces can be cultivated through place making (Johnson et al. 2018; Tan et al. 2018). The simple provision of benches for example has been found to increase a person’s time spent in nature and facilitates interaction with nature such as through birdwatching and quiet reflection (Bell et al. 2018; Palliwoda and Priess 2021).

Closely related to the *aesthetics* domain is the domain of *paths and trails* as they are necessary to facilitate access within wetlands, and to some vantage points (this covered under the *aesthetics* domain). Concerningly, however, our results of low scores in relation to *paths and tails* indicate that access to explore and experience Tasmanian wetlands is highly constrained (28% on average, with only 6 wetlands ranking moderate to high). If the accepted global policy intention is to encourage people to visit wetlands, numerous studies have noted that the provision of paths and trails is essential to facilitate such visitation (Schipperijn et al. 2010; Finlay, et al. 2015; Bell et al. 2018). Also, notably, potential visitors to Tasmanian wetlands have identified trail walking as their most preferred recreational activity and walking tracks as the most desired on-site facility (Wang et al. 2022).

Wetland visitors also require a wider range of *facilities and amenities*, these captured under its own domain in WWT. Our results for this domain are surprisingly higher (37% on average) than the scores for *paths and trails* (Table 4). This is likely attributable to a fortunate coincidence in that many of our wetlands that scored high in this domain were near other, more popular environments such as beaches and campgrounds that have toilets, bins, picnic tables, play areas, and other visitor amenities. It is known that such amenities are linked to increased visitor usage, positive experience, and time spent in natural areas (Bell et al. 2018; Palliwoda and Priess 2021). Also, these broader set of facilities and amenities cater for different types of visitors like older people (e.g. seating) and children (e.g. structured play opportunities) (McCormack et al. 2010).

#### Natural values/features

Wetlands are foremost areas that harbour and support natural values, and the conservation and restoration of these values have been recognised as important in the context of promoting HNC in wetlands (Pratt and Prahalad 2025). The *natural features* domain addresses the need for this balance, with results suggesting that it is not currently being achieved. Most wetlands lacked infrastructure designed to protect the natural values present within them, reflected in a low average score of 34% and only five sites scoring moderate to high. Improving scores in this domain is especially important in wetlands where visitation levels are high, or where wetlands are promoted for visitors, such that protective infrastructure like signage and fencing can be provided to protect natural values (Tang 2015; Irazábal 2018; Yuxi and Linsheng 2020). The balance here is the provision of visitor infrastructure that facilitates use (e.g. tracks, toilets) while also protecting the environment (e.g. signs, fences) (Irazábal 2018). More generally, if increasing visitation to wetlands is a desired goal, there needs to be a clear understanding where human footprint can be directed such that the impacts are compatible with a wetlands’ carrying capacity and conservation needs (O’Rielly 1986; Boschken 2013; Mexa and Coccossis 2017). This is precisely the role we expect WWT can play in identifying suitable sites within a region that can be developed with increased visitation as a goal.

Also, the lack of signage with information about the natural values in the wetlands we assessed represents a missed opportunity to strengthen support for wetland conservation. Educational signage has been shown to increase public awareness of natural values and visitors’ ability and willingness to protect natural values (Bradford and McIntyre 2007; Donnelly et al. 2021). Furthermore, Wilson and Tisdell (2005) highlight the crucial role education has for the conservation of poorly known and threatened species, both of which are present in wetlands given their high levels of biotic diversity. In the wetlands being developed and promoted for visitation, signage of natural values thus becomes a key consideration for positive HNC-derived conservation gains.

#### First Nations site heritage and values

Aboriginal and Torres Strait Islander peoples have long been excluded from Australian history books, the national flag, the anthem, and for many years, from Australian democracy (Stanner 1968; Reconciliation Australia 2024). Regrettably, our results for this domain paint a bleak picture, indicating that such exclusion remains prevalent. Only one site, Tamar Island Wetlands, scored fair or above, with a low average score of just 2% across all sites assessed. This represents a missed opportunity, as potential visitors to Tasmanian wetlands have expressed a strong interest in Aboriginal cultural interpretation as part of their experiences (Wang et al. 2022). Efforts to improve scores across this domain must ensure that both tangible and intangible First Nations heritage and values are acknowledged and protected with respect to best practices concerning Aboriginal and Torres Strait Islander people's engagement, partnership and self-determination (Janke et al. 2021; McConnell et al. 2021).

### A (near) perfect wetland for HNC: Tamar Island Wetlands

**Fig. 5:**
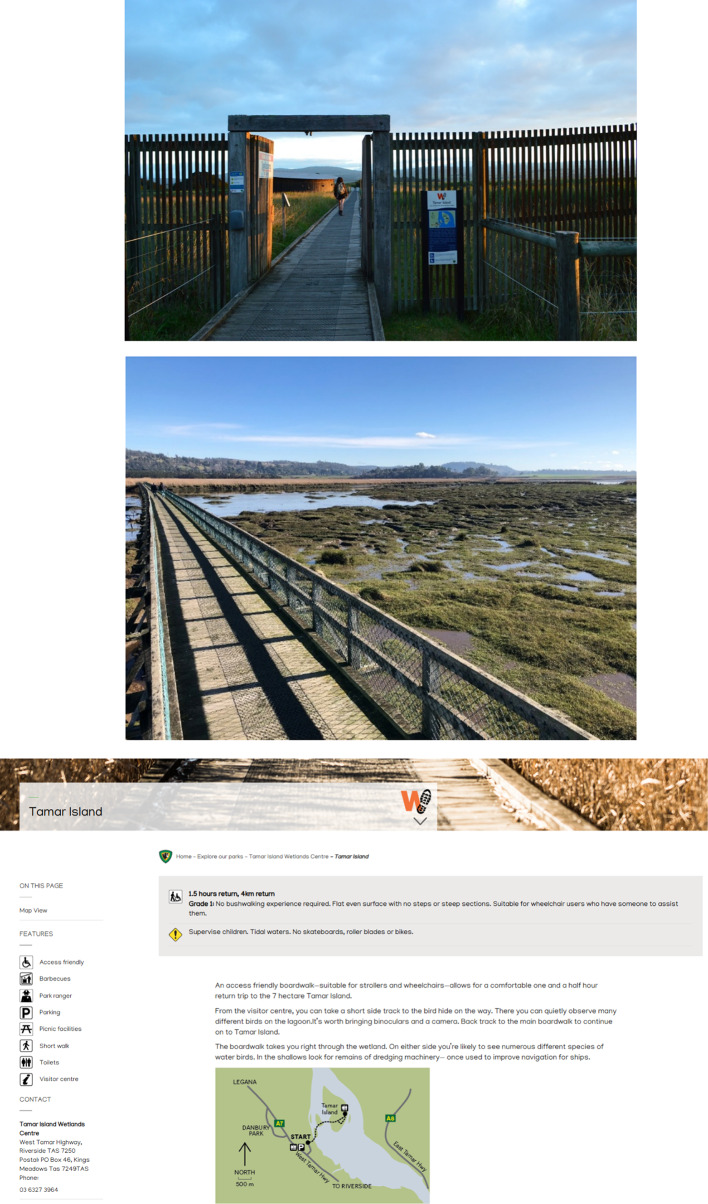
Top image: The entrance the popular Tamar Island Wetlands near Launceston, the second largest city in Tasmania. The site provides visitors with amenities like paths and trails, bird hides, vantage points, benches and rubbish bins, and has a small visitor information centre with views over wetlands, interactive displays, a shop and toilet facilities. Middle image: A key feature of the site is its access (with extensive boardwalks) and accessibility (e.g. potential for pram and wheelchair use). Bottom image: screenshot of web-based information available to promote Tamar Island Wetlands. Source: Top/Middle images supplied by authors; Bottom image obtained from Tasmania Parks and Wildlife Service website

Although our results as discussed above largely indicate areas where HNC can be strengthened, they also reveal examples of best practice that can be used to inspire and inform future efforts to enhance HNC in wetland environments. Notably, among the 21 wetlands assessed in this study, the Tamar Island Wetlands (Fig. 4, K) was the only one ranked as High with an average score of 77% (Table 4; Fig. 5). As such, it provides a model for other wetlands in being able to foster HNC in wetlands. Obviously not all wetlands need to score as highly as Tamar Island Wetlands given varying objectives (e.g. some wetlands are nature reserves where public visitation is limited for good reasons). However, at a local or regional level, a policy goal could be to have a wetland that is set up as Tamar Island Wetlands is, with specific regard to its visitors’ centre which encourages a broad demographic to learn about wetland values and threats. This enables people to develop and strengthen their knowledge of and connection to wetlands, particularly for those unfamiliar with wetlands (Wang et al. 2022). This underscores the contribution that the new WWT seeks to make – in providing an inventory of HNC at a suitable scale that can inform the development of a network of sites with varying affordances towards fostering HNC.

## Conclusion

This study has outlined the development and first use of the WWT, a tool for assessing HNC in wetlands. The review undertaken by this study as a part of the development stage of WWT has highlighted the lack of current tools available for assessing environmental attributes/physical assets for HNC in blue/wetland environments globally and in Australia. The new WWT addresses this gap, providing a method via which wetlands can be inventoried in terms of the current *orientation* and *access* available to visitors, and therefore current opportunities which may enhance HNC in wetland areas. Proof-of-concept testing of the tool has revealed an inventory of opportunities for HNC in wetlands across Tasmania driven by *orientation* and *access.* Discussion of the WWT assessment has revealed that the tool can provide important insights which can be used to plan and implement provisions for the enhancement of HNC. The tool can be used at a single site or can be used as a region-wide assessment, such as by local councils, to inventory their wetlands for provisioning HNC opportunities. Thus, the tool addresses a huge gap in understanding what kinds of experiences wetlands are currently able to support based on their existing features.

## Supplementary Information

Below is the link to the electronic supplementary material.

Supplementary file1 (PDF 800 KB)

Supplementary file2 (PDF 3292 KB)

Supplementary file3 (XLSX 257 KB)

Supplementary file4 (PDF 220 KB)

## Data Availability

All data supporting the findings of this study are available in the supplementary material 3 accompanying this article.
