# Supplementary material for: Assessing human–nature connection: A systematic review and a new Wetland Wanderer Tool for auditing nature connection in wetland environments
Source: Ambio. 2026 Feb 20;55(8):1933–53. doi: 10.1007/s13280-025-02335-1 (PMC13319621; doi:10.1007/s13280-025-02335-1)
Supplement: Supplementary file 1 — Supplementary file1 (PDF 800 KB) [file 13280_2025_2335_MOESM1_ESM.pdf]

Supplementary Material 1 (Tables S1 to S8) for

Assessing human-nature connection: A systematic review and a new Wetland Wanderer Tool for auditing nature connection in wetland environments

Kate Pratt [AB], *Vishnu Prahalad* [A]

[A] School of Geography, Planning and Spatial Science, University of Tasmania, Hobart 7001, Tasmania, Australia

[B] Corresponding author. Email: kate.pratt@utas.edu.au

July 2024

Table S1. Results of systematic literature review including (1) publication time, journal, location of development AND (2) aim and field of research, intended environment for use, and intended users.

Tool	Author	Year	Location	Journal or other affiliation	Aim	Field	Intended Users	Environment
BEAT	Mishra et al.	2020	Europe	Urban Forestry & Urban Greening	Assess quality of blue spaces to give better reference for planners/designers in providing health-promoting affordances	physical activity; health and well-being	Landscape planning and environmental management professionals; community groups	Urban blue space
BRAT-DO	Bedimo-Rung et al.	2006	USA	Journal of Physical Activity and Health	Assess park characteristics for better understanding of their use for physical activity.	Physical activity	Not outlined	Urban parks
CPAT	Kaczynski et al.	2012	USA	American Journal of Preventative Medicine	Enable diverse stakeholders to audit community parks quickly and reliably for their potential to promote physical activity.	Physical activity	Community stakeholders	Urban/ suburban parks
EAPRS	Saelens et al.	2005	USA, Kansas City	Journal of Physical Activity and Health	Measure environments in which physical activity could occur. Characterise the physical environments within public parks and playgrounds.	Physical activity	Not outlined	Urban/urban periphery/suburban parks and playgrounds
MexPOS	Medina et al.	2022	Mexico City, Mexico	International Journal of Environmental Research and Public Health	Assess public open space features within the Mexico City context so they can be modified/created to increase attendance and healthy lifestyles.	Physical activity; health and wellbeing	Administrators, city planners, urbanists and stakeholders.	Urban public open space
NEST	Gidlow et al.	2018	Europe	Urban Forestry & Urban Greening	Measure the quality of natural environments in different European cities and their health promoting attributes.	Quality of urban green/blue space	General purpose (no expertise required)	Urban green and blue space
PARA	Lee et al.	2005	USA, Kansas City	International Behavioural Nutrition and Physical Activity	Assess physical activity resource quality	Physical activity;	Not outlined	Urban neighbourhoods
PARCS	Perry et al.	2018	New Zealand	Disability and Health Journal	Assess the accessibility and usability of public parks and playgrounds	Disability	Not outlined	Urban public parks and playgrounds
PARK	Bird et al.	2015	Canada, Montreal	BMC Public Health	Assess parks for appropriateness for physical activity for youth	Physical activity; physical health; youth	Not outlined	Urban parks
PEAT	Troped et al.	2006	USA, Massachusetts	Journal of Physical Activity and Health	Assess trail/path characteristics for physical activity	Physical activity; health and well-being	Researchers	Urban/suburban/rural paths/trails
POST	Broomhall et al.	2004	Australia, Perth WA	University of Western Australia, Centre for the Built Environment and Health	Assess public open spaces such as parks and ovals, with particular emphasis on the physical attributes that may either encourage or discourage their use for physical activity.	Physical activity	Researchers	Public open space
SAGE	Byrne et al.	2005	USA, Los Angeles	The Green Visions Plan	Assess the current distribution of park, recreation, open space, and beach-related assets so that future planning is provided direction.	Planning	Los Angeles metropolitan region	Green-space environments
SPEAK	Hyung Sook Lee	2022	Korea	Landscape and Urban Planning	Assess park quality for Korean seniors	Physical activity; accessibility for aging population	Government staff; community member (no special skills required)	Urban parks
WIAT	Thompson & Roe	2010	Scotland	Forestry Commission Scotland	Aimed at improving woodlands so as, ultimately, to improve people's quality of life and to allow evaluation of change over time in the same woodland.	Research	Not outlined	Wood in and around towns

Table S2. Results from systematic literature review including (3) supporting material, development strategy, reliability/validity, problems in reliability and results/findings.

Tool	Development strategy	Reliability/ validity	Problems in Reliability	Supporting Material	Results/findings
BEAT	Developed using results from review of existing tools; adjusted to fit conceptual framework; 2 stages of testing (stage 1: one common and several expert assessors rated 16 sites independently. Stage 2: two assessors rated 21 sites independently)	Assessment scores of the primary assessor used to create a proxy standard for all sites. Inter-rater reliability (inter-class correlation coefficient) stage 1& 2. Intra and inter-site comparisons (stage 1:6 sites; stage 2: 18 sites). Kappa coefficients used to assess agreement.	Subjective elements have less reliability than non-subjective elements	Guidance provided	Two assessors recommended; results should be pooled to test inter-rater reliability (kappa). With good guidance and training reliability increases.
BRAT-DO	Developed with an expert panel via Delphi method; conceptual framework; three rounds of testing tool refined after each round (round 1: one data collector 400-acre park; round 2: same park 16 teams of observer pairs; round 3: 15 teams of observer pairs trained; 7 teams sent to original park, 8 sent to 200 acre park).	Gold standard to test validity (round 3: two investigators closely involved in development assessed each area by consensus); Inter-rater and domain validity by comparing results obtained by fieldworkers with gold standard;.inter-observer agreement by percent agreement to test reliability.	Percent agreement overestimates true agreement as it does not account for agreement due to chance. Subjective elements less reliable. Reliability only tested in single location.	Guidance manual available	Training is important and should involve classroom time and field practice. Maps of the area and training on their use is helpful to make sure elements are not missed or mistaken
CPAT	Six stages of development - stage 1: Review of existing tools; stage 2: focus group and workshop with stakeholders; stage 3: three key informant interviews with researchers familiar with auditing tools; stage 4: tool reviewed and tested in single park by stakeholders; stage 5: tested again in 59 parks (as Kansas City has 219 parks this is to include all diversity); stage 6: final workshop to gain feedback on stakeholder experience.	Inter-rater reliability could not be measured	Percent agreement is more appropriate when little variability exists among rating (>70% for this study). Subjective or temporally variable elements have low reliability.	Guidance manual available	User-friendly audit tools should be 2-8 pages or 15-60 minutes long. They should have simple question response formats, space for subjective comments and directions within the tool that are easy to follow and require minimal training.
EAPRS	Park and recreation professionals (34) and frequent park users (29) surveyed about park and playground elements and qualities. Results guided inventory selection. 92 observations made in parks and playgrounds. Instrument revised following inter-rater reliability testing. Final observation in 21 parks and 20 playgrounds final revision and reliability testing.	Inter-rater reliability (kappa; ICC; Percent agreements where kappa/ICC not appropriate i.e.where no response variability exists)	Subjective items less reliable, temporal nature of some items thought to effect low reliability also. Items with low variability could not be tested effectively for reliability.	Guidance manual available	Reliability should be tested in each park specific context.
MexPOS	Meeting with specialists in physical activity, nutrition, urbanism, architecture, and public policy (*n* = 13) in which characteristics of POS that could foster physical activity were discussed and listed. 3 parks visited where characteristics were verified, 16 items created. This was compared and complemented with items from BRAT-DO and EAPRS. 20 items from BRAT-DO included, and 12 items from EAPRS added. Items and answers adapted for Mexican context and from specialist group. Urban planners pilot tested new version in 1 park, adapting it. Two volunteers from the research group applied the tool and gave feedback on clarity of instructions. Tested in 944 public open spaces across Mexico City	Exploratory factor analysis (validity of items and the relationship between subscales).	Only tested in a single city. More research needed to establish inter-rater reliability and validity.	Not available; training used in development	EFA an appropriate measure due to high corroboration in inventory determination
NEST	Review of existing tools; NGST augmented through expert consultation; items added from POST; CABE (not reviewed in this study) and BRAT-DO; data collected in 4 cities (290 audits; 19 assessors; 174 natural environments; 4 European cities); domain reliability and sense check; items added, amended, or removed for final tool.	Domain reliability (Cronbach's alpha coefficient and inter-item correlations); inter-rater agreement (Pearson's correlations)	ICC not used as not same two assessors completed all audits. Typologies developed were not able to be tested in each city.	Not available; training used in development	'fitness for purpose' scores environments based on typology therefore can be used for a range of environments, specifically more so than those developed to assess physical activity.
PARA	Internet and telephone book searching conducted to identify physical activity resources in neighbourhood using a list of terms identified in previous study. All resources mapped and verified, then trained assessors conducted windshield surveys to confirm locations of resources and find others not identified. Trained field coders assessed each resource, data collectors counted and coded 25 elements of each resource. Instrument pilot tested and revised numerous times to achieve final form (information regarding method of development could not be found).	Inter-rater reliability	Reliability testing not mentioned	Guidance manual provided	Incivilities were shown to be important in distinguishing between neighbourhoods, those in low-income areas had higher incivility scores than those in high income areas. Thereby showing merely building a park in a deprived area may in insufficient for insuring its intended use and maintenance.
PARCS	Developed from literature review of existing tools which adapted existing tools based on recommendation from NZ standard guide for buildings and associated facilities and other guides. Also developed in consultation with other stakeholders in disability action (reviewed and modified). Two researchers than conducted independent piolet evaluations in two parks, then reconvened to discuss evaluations, consensus reached, minor amendment made.	Inter-rater reliability (kappa)	PARCS tool is not a fully validated tool and requires further psychometric testing.	Not available; training used in development	No parks evaluated meet nation standards and/or international guidelines for park and playground design.
PARK	Developed from literature review of existing tools. Items from POST and BRAT-DO modified and adjusted for PARK. BRAT-DO framework applied. Piloted among observers from diverse ethnic backgrounds in early twenties. Observer training occurred over 9 days.	Inter-intra-rater reliability (kappa) of items. Inter-rater reliability tested in AUS were compared (POST); test-re-test.	PARK tool aims to assess features of parks that are conceptually attractive for youth, this has not yet been validated. Subjective items less reliable. Large time difference between tests.	Not available; training used in development	Assessment should be carried out at the same time of day on the same day of the week. Items tested in Montreal and Perth (PARK; POST) likely generalizable to other urban environments.
PEAT	Literature review of parks and recreation, landscape architecture, transportation, and planning literatures to identify environmental characteristics of trails that have been documented to influence use. As a second strategy, we developed and implemented a brief intercept survey with a convenience sample of 73 adult trail users at two study sites during the spring of 2003. Thirdly, solicit input from our transdisciplinary team. Pre-tested on 43 trail and intersecting road segments at two study sites. Amended.	Gold standard (using GIS informed criterion of ten trail design characteristics or amenities); inter-rater reliability (kappa; ICC)	Low reliability for subjective items	Guidance manual provided	First study to test both reliability and validity of an audit tool for community and trails and paths.
POST	Developed in 1996 as a part of the SEID I project and since adapted. In-depth interviews and a Delphi process, conceptual frameworks were developed to examine the physical environmental factors relevant for walking for recreation and for transport. These factors were arranged into three levels: features (overall factors that influence the physical environment), elements (factors that are components of the features) and items (factors that have the potential to be changed to improve an element).	Inter-rater reliability (kappa). Weighted: the items, elements and features were weighted according to average weights provided by the Delphi participants; specifies different scoring for different groups in fact sheet.	Guidance manual must be used	Guidance manual available; fact sheet on SEID I project findings	Manual must be used for clarification to increase reliability
SAGE	Adapted from other tools (SPACES and ROUTES). Other information regarding information not outlined.	Tools adapted from review have had inter-rater reliability testing	Unreliable if not familiar with materials used	Guidance manual available	Only manual is available

SPEAK	Initial assessment items from review of existing tools. Incorporation of other items pertinent to older people based on lit review of environmental factors influencing seniors park use and PA. experts scrutinised list of assessment items. Piloted audit in 42 parks across 4 districts. After the pilot study was conducted and input from the evaluators was collected, several items and response scales were modified for additional clarity.	Inter-rater reliability (kappa)	Subjective items least reliable. To minimise variability and increase reliability 5-point scale rescaled to dichotomous or 3-point scale	Training required no guidance manual available	Not as detailed or comprehensive as other auditing tools because it was developed to be used by those without expertise in research field.
WIAT	Adapted from previous auditing tools. Information regarding exact development process not available.	Robustness is ensured by testing the tool in a range of circumstances with a range of auditors, to ensure it comes up with consistent and credible results.	Training needed to ensure reliability; assess in pairs	Guidance manual available	Only manual available

Table S3. Results from systematic literature review including (4) format of the tool, length of the tool and, scoring methods employed; AND (5) domains as outlined by tool.

Tool	Format	N. of items	Scoring	Domain names (number of domains)
BEAT	Free online access	125	Presence/absence; 5-point Likert scale; multiple choice checklist; comments; observations.	Environment; aesthetic; social; physical (4)
BRAT-DO	Pen and paper	181	Presence/absence; 5-point Likert scale; categorical response; written information.	Features; condition; access; aesthetics; safety (5)
CPAT	Pen and paper	28	Presence/absence; rating scale; categorical response; comments	Park information; access and surrounding area; park activity areas; park quality and safety (4)
EAPRS	Pen and paper (new format eCPAT was not reviewed for this study)	751	Presence/absence; 5-point Likert-type scale	Paved and unpaved trail and path items; designated specific use and water items; amenities and facilities; play equipment and field and court items. (4)
MexPOS	Pen and paper	131	Likert scales; binary scales; ordinal scales	General park information; food environment and health wellness; maintenance; amenities; signalling; safety; perceived environment; urban surroundings. (8)
NEST	Excel (new format eNEST not reviewed for this study)	47	Rating scale; binary scale; ordinal; domains weighted to determine different typology specific overall scores.	Accessibility; recreation facilities; amenities; aesthetics-natural; aesthetics-non-natural; incivilities; significant natural features; usability (8)
PARA	Pen and paper	49	Rating scale	Location; type; cost; features; amenities; quality; incivilities (7)
PARCS	Pen and paper	80	Rating scale; binary scale	Accessible routes (parking spaces; path surfaces); facilities and amenities (play areas; rest areas; restrooms; drinking fountains) (2)
PARK	Pen and paper; excel	92	Presence/absence; binary scale; rating scale	Activities; environmental quality; services; safety; general impression (5)
PEAT	Microsoft Access form	36	Rating scale; binary scale; categorical response; ordinal scale.	Design features; overall park or trail characteristics; aesthetics; “human” environmental factors; “situation” characteristics or neighbourhood contextual variables. (5)
POST	Pen and paper	49	Rating scale; multiple choice checklist; binary scale	Activities; environmental quality; amenities; safety (4)
SAGE	Desktop; web-based data entry ArcPad GPS; pen and paper	18	Rating scale; binary scale; multiple choice checklist	Facilities and amenities; landscape features; condition; safety (4)
SPEAK	Pen and paper	36	Rating scale; binary scale	Access; amenities; safety/incivilities; aesthetics; recreation facilities (5)
WIAT	Pen and paper	23	Composite	The neighbourhood; access/signage to woodland; woodland quality; facilities; use; maintenance/management (6)

Table S4. List of groups and experts consulted with, and activities undertaken as a part of the development of the Wetland Wanderer Tool (WWT).

**Name of group, expert or activity**	**Contribution to WWT**	**Contact**
National Environmental Science Program (NESP), project IP1.02.01 - Nature Connection.	Regular meetings held by the group required the lead author to provide updates on progress of the tool and receive feedback from others in the group.	emily.flies@utas.edu.au
Vanessa Adams:<br>Associate Professor, Conservation and Planning<br><br>Doctor Emily Flies:<br>Lecturer in Environment and Sustainable Communities; NESP coordinator	Provided insight into any relevant tools/ methods used for assessing nature connection and what they thought of the proposed WWT.	vm.adams@utas.edu.au<br><br>emily.flies@utas.edu.au
Doctor Jason Byrne: Professor of human geography and planning at the University of Tasmania. Co-developer of SAGE.	Email correspondence regarding any known tools of relevance.	jason.byrne@utas.edu.au
Australasian Mangrove and Saltmarsh Network Conference 2023.	Oral presentation given by author (K. Pratt) concerning the results of this paper. Feedback received.	Corresponding author.

Table S5. Initial draft (Version 1) of WWT comprising 15 domains and 76 items. This version was tested in Adelaide to highlight revisions required towards developing Version 2.

**Domain**	**Item**
Site Context	1. Name of site<br>2. Location of site<br>3. Can the site be located via a search engine (e.g., google) or map application (e.g., Apple Maps)?<br>4. Can information promoting recreation in the area be found on social media platforms? Protection/historical/cultural status:<br>5. Is there a brochure or other information product (e.g., map) available (online or on- site)?<br>6. Date/time/weather
Education and Outreach	7. Are there any events advertised (Are there any events advertised (online or on-site)? (circle correct answer) YES / NO<br>8. Are there any volunteer programs advertised (e.g. citizen science) (online or on-site)? (circle correct answer) YES / NO<br>9. Are tours of the area available? (circle correct answer) YES / NO<br>10. Is there a visitors' centre? (circle correct answer) YES / NO Please specify what is in the visitor centre:<br>11. Are there signs concerning wetland values and biodiversity? (circle correct answer) YES/ NO<br>12. Is there a Friends of Group attached to this area or some other form of volunteer enterprise? (circle correct answer) YES / NO
Use of Site	13. Are there any people at the site? (circle correct answer) YES / NO<br>14. How many people? 0. <10 >10 >50<br>15. List activities you can see people undertaking.

	16. List activities you can see indirect evidence of (e.g., event times).
Signage	17. Are there signs at the site? (circle correct answer) YES / NO (go to ACCESS) IF YES circle all that apply<br>a. EDUCATION<br>b. INFORMATION<br>c. HAZARD<br>d. PROHIBITION<br>e. IF OTHER please specify here:<br>18. What condition are the signs in? (circle correct answer)<br>a. Education POOR FAIR GOOD<br>Information POOR FAIR GOOD<br>Hazard POOR FAIR GOOD<br>Prohibition POOR FAIR GOOD<br>Other POOR FAIR GOOD<br>19. What message are the signs conveying? Please specify here:<br>20. List activities which are prohibited:<br>21. List activities which are allowed:
Access	22. Is there any indication of the site's existence from the nearest main road? (circle correct answer) YES / NO<br>Please specify how this is conveyed:<br>23. Can you enter the site? (circle correct answer) YES / NO IF NO please specify:<br>24. How many entry points are there?<br>25. Is there parking available? (circle correct answer) YES / NO<br>26. Is the parking specifically for visitors? (circle correct answer) YES / NO<br>Is public transport available directly to site? (circle correct answer) YES / NO<br>27. Is bike storage available? (circle correct answer) YES /NO

Accessibility	28. Is the site accessible to all levels of ability? (circle correct answer) YES / NO IF NO please specify:<br>29. Are there any specific measures the site has taken to ensure accessibility for everyone (circle correct answer) YES / NO IF YES please specify:<br>30. Does the site have signage in languages other than English? YES / NO
Safety and Security	31. Are there any hazards not accounted for by management? (circle correct answer) YES/NO IF YES please specify:<br>32. How far away is the nearest emergency service?<br>33. Is there mobile phone reception? YES/NO (please specify provider)<br>34. Is anyone else at the site? (circle correct answer) YES / NO<br>35. Has anyone been injured at the site during the last year? (circle correct answer) YES/NO IF YES please specify how:<br>36. Do you feel safe? (circle correct answer) YES / NO IF NO Please specify why:
Incivilities	37. Is there litter present? (circle correct answer) YES/NO IF YES please specify the amount:<br>38. Is there evidence of vandalism? (circle correct answer) YES/NO IF YES please specify what:<br>39. Is there evidence of anti-social behaviour? (circle correct answer) YES/NO IF YES please specify what:
Visual Aesthetics	40. Are there any viewing platforms in the site? (circle correct answer) YES/NO<br>41. What is the quality of views? (circle correct answer) POOR FAIR GOOD Please specify:
Non-visual Aesthetics	42. Are there any pleasant smells? (circle correct answer) YES / NO<br>43. Are there any unpleasant smells (circle correct answer) YES / NO<br>44. Are there any pleasant noises? (circle correct answer) YES / NO

	45. Are there any unpleasant noises? (circle correct answer) YES /NO
Paths/Trails	46. What is the condition of the path/trail? (circle correct answer) POOR FAIR GOOD<br>47. What is the path/trail material (e.g., board walk; gravel)?<br>48. How extensive is the path/trail?<br>49. What is the trail grade?<br>50. Is the trail steep?<br>51. Are there stairs?<br>52. Is there information regarding walking/cycling opportunities? (circle correct answer) YES/NO Please specify how this information is conveyed:<br>53. Are there signs along the path/trail? YES/NO Please specify
Facilities/ Amenities	54. Is there a visitor centre? (circle correct answer) YES/NO Please specify what is inside:<br>55. Are there toilets? (circle correct answer) YES/NO What is their condition? POOR FAIR GOOD<br>56. Are there benches? (circle correct answer) YES/NO<br>57. What is their condition? POOR FAIR GOOD<br>58. Are there barbeques? (circle correct answer) YES/NO<br>59. What is their condition? POOR FAIR GOOD<br>60. Are there picnic tables? (circle correct answer) YES/NO<br>61. What is their condition? POOR FAIR GOOD<br>62. Are there trash cans? (circle correct answer) YES/NO<br>63. What is their condition? POOR FAIR GOOD<br>64. List any other facilities/amenities on site and their condition:
Maintenance/ Management	65. What is the current overall condition of amenities/facilities? (circle correct answer) POOR FAIR GOOD<br>Please justify:

	66. Is there evidence that the site is being managed? (circle correct answer) YES / NO Please specify what and by whom:
Cultural/ Historical features	67. Is there any information relevant to cultural/historical elements? (circle correct answer) YES /NO<br>Please specify how this information is conveyed:<br>68. Are there any cultural/historical features within the site? (circle correct answer) YES / NO IF YES what?
Natural Features	69. Is vegetation native? (circle correct answer) YES / NO<br>70. Is the site natural? (circle correct answer) YES / NO<br>71. Has the site been landscaped (circle correct answer) (circle correct answer) YES/ PARTIALLY/ NO<br>72. Is there information regarding flora/fauna of site? (circle correct answer) YES/NO IF YES how this is conveyed<br>73. Is there any information regarding threats to the area? (circle correct answer) YES/NO IF YES how is this conveyed?<br>74. Have restoration activities been undertaken at the site? (circle correct answer) YES/NO<br>75. Is access impacting habitat protection? (circle correct answer) YES / NO Please explain<br>76. List the types of animals you can see direct/indirect evidence of:

Table S6. Wetland Wanderer Tool Scoring Sheet. Numbers in red colour text indicate the number of points given to each question. If the section is blank, it means there are no points allocated for answers to those questions. Note there are some inconsistencies in the data (number of items in the tool used across 21 wetlands) which reflect minor updates made to the tool after the initial scoring. The sheet provided is a revised version, which includes adjustments to the number of items. However, the scoring itself was based on the earlier version of the tool. These differences do not affect the interpretation of results but reflect the tool's ongoing refinement during its development.

ONLINE PRESENCE	
Name of Site	
Type of wetland	
Search the below phrases in reference to the area the site is located:<br>- Tourism sites...<br>- Tourism places...<br>- Tourism attractions...<br>- Tourism spots...<br>Do the top five webpage results from each search note this wetland as a place to recreate?	0 1 2 3 4<br>0 +1 +2 +3 +4
Search the following phrase followed by the wetland's name:<br>- Volunteer opportunities...<br>- Volunteering...<br>- Volunteer work...<br>- Places to volunteer<br>Do the top five webpage results from each search note current	0 1 2 3 4<br>0 +1 +2 +3 +4

volunteering opportunities? IF YES ADD 1 3places	
Protection status (e.g., Ramsar Convention; IUCN; National Reserve System)	
Cultural Heritage status (e.g., World Heritage)	
First Nations Heritage status	
	TOTAL SCORE /8

EDUCATION AND OUTREACH		
Are there volunteering opportunities advertised onsite	YES +1	NO
Are there any events advertised?	YES +1	NO
Are tours of the site available?	YES +1	NO
IF YES are the tours focused on wetland values and biodiversity?	YES	NO -1
Is there a Visitor Centre attached to the site? IF	YES +1	NO

YES does the centre have a focus on information specific to wetlands?	YES	NO -1
	TOTAL SCORE /4	

USE OF SITE		
How many people are onsite?	0 +0 <10 +0.25 10-30 +0.5 30-50 +0.75 50+ +1	
What activities are people undertaking?	Bird watching +0.16<br>Trail walking<br>Photography<br>Camping<br>Fishing<br>Hunting	Picnic/BBQ<br>Bike riding<br>Kayaking<br>Boating<br>Volunteering<br>Working
If people are undertaking activities not listed above, please indicate what they are here		
	Bird watching +0.16	Picnic/BBQ

What activities can you see indirect evidence of (e.g., signs; campground)	Trail walking<br><br>Photography<br><br>Camping<br><br>Fishing<br><br>Hunting	Bike riding<br><br>Kayaking<br><br>Boating<br><br>Volunteering<br><br>Working
If there is evidence of activities not listed above, please indicate what they are here		
	TOTAL SCORE /4	

Condition should be noted as FINE; OBSTRUCTED -1; DAMAGED -1; ILLEGIBLE -1

SIGNAGE			Condition
Is there signage at the site's boundary indicating its presence?	YES +1	NO	
Is there signage within the site? (IF NO please move to ACCESS)	YES +1	NO	*Leave blank*
Does signage contain educative information about natural values and biodiversity?	YES +1	NO	

IF YES is the information specific to wetland values and biodiversity?	YES +1	NO	*Leave blank*
Does signage contain information outlining what activities are prohibited onsite?	YES +1	NO	
Is there signage which indicates who the tradition owners of the site are?	YES +1	NO	
Is there signage with a map of the site?	YES +1	NO	
Is there signage with information regarding who manages the site?	YES +1	NO	
Is there signage with information regarding volunteer opportunities?	YES +1	NO	
Is there signage encouraging Citizen Science? (e.g., *iNaturalist*)	YES +1	NO	
If there are other forms of signage, please indicate what information they relay			

	IF CONDITION IS ILLEGIBLE -1<br>IF CONDITION IS DAMAGE/OBSTRUCTED -0.5<br>TOTAL SCORE /10

**ACCESS**		
Is there any indication of the site's existence from the nearest main road?	YES +1 PARTIALLY +0.5 NO	
Can you enter the site?	YES +1	NO
IF NO is there a designated point along the road to view the site?	YES	NO -1
Is car parking available?	YES +1	NO
IF YES is parking specifically for visitors to the site?	YES	NO -1
Is parking less than 1km from the site?	YES +1	NO
Is the road to the site sealed?	YES +1	NO
IF NO is the road to the site FWD access only?	YES -1	NO
Is public transport available <1km from the site?	YES+1	NO

Is bike riding allowed onsite?	YES+1	NO
IF NO is there a bike rack?	YES	NO-1
	TOTAL SCORE /7	

**ACCESSIBILITY**					
Is there disability parking available?	YES +1		NO		
Is the site accessible for people with vision impairment? (e.g., braille on signs)	YES +1		NO		
Does the site have signage in languages other than English?	YES +1		NO		
IF YES which languages?					
If there are no trails within the site move to SAFETY/SECURITY					
Are there stairs along the path/trail	YES		NO +1		
IF YES how many?	1-10	10-20	20-50	50-100	100+
Does the trail have uphill sections?	YES		NO +1		

Is the path/trail smooth, slip resistant and free from uneven surfaces?	YES +1 PARTIALLY +0.5 NO	
Does the path/trail have a width of 1m or greater at all times?	YES +1 PARTIALLY +0.5 NO	
IF PARTIALLY for either of the above two questions are these sections located at the entrance point and do they enable access to facilities/amenities	YES	NO -1
IF YES what facilities/amenities		
	TOTAL SCORE /7	

SAFETY AND SECURITY		
Are there any hazards listed on signage onsite?	YES +1 PARTIALLY +0.5 NO	
Are there any hazards you can see onsite not noted on signage?	YES -1	NO
Is there mobile phone reception?	YES +1 PARTIALLY +0.5 NO	
What provider do you use?		

Is anyone onsite exhibiting anti-social behaviour? (e.g., intoxication; loitering)	YES		NO +1
IF YES what are they doing?			
Do you feel safe?	YES +1		NO
			TOTAL SCORE /3
If there are no trails within the site move to INCIVILITIES			
Are trails within the site enclosed by vegetation? (e.g., can you see houses, the road, and/or exits?)	YES	PARTIALLY +0.5	NO +1
Are the trail edges clearly defined? (e.g., is the vegetation along the edges overgrown and/or containing rubbish?)	YES +1	PARTIALLY +0.5	NO
			TOTAL SCORE /6

**INCIVILITIES**			
Is there litter present?	YES		NO +1
IF YES please indicate the amount	LOW	MODERATE -0.5	HIGH -1

Is there evidence of vandalism? (e.g., human caused damage)	YES	NO +1
IF YES please indicate what vandalism has occurred		
Is there any evidence of alcohol or drug use?	YES	NO +1
Is there any sex paraphernalia present? (e.g., condoms)	YES	NO +1
	TOTAL SCORE /4	

AESTHETICS		
Can you see the wetland from the nearest road?	YES +1	NO
Is there a designated place to view the wetland from the road?	YES	NO -1
IF YES is the view impeded? (e.g., buildings; vegetation)	YES -1	NO
IF YES what is it impeded by?		
Is there a place to sit and view the wetland?	YES +1	NO
TOTAL SCORE /2		
If you cannot access the site please move to PATHS/TRAILS		
Can you see the wetland **within** the site?	YES +1	NO

Are the views impeded by anything? (e.g., buildings; vegetation)	YES -1	NO
IF YES what are they impeded by?		
Is there a place to sit and view the wetland?	YES +1	NO
Are there any pleasant smells? (e.g., flowers)	YES +1	NO
Are there any unpleasant smells? (e.g., rubbish)	YES -1	NO
Are there any pleasant noises? (e.g., birds; water)	YES +1	NO
Are there any unpleasant noises? (e.g., cars; machinery)	YES -1	NO
	TOTAL SCORE /6	

PATHS AND TRAILS		
Are there trails/paths around/within the site? (IF NO move to FACILITIES/ AMMENITIES)	YES +1	NO

Is there a sign indicating where paths/trails lead?	YES +1	NO
Is the path/trail material consistent throughout?	YES +1	NO
What is the path/trail material? (circle all that apply)	Dirt<br>Gravel<br>Sand	Board walk<br>Concrete<br>Other:
Are there places to sit along the path/trail?	YES +1	NO
IF YES are these places located within 50m of each other?	YES +1	NO
Are there signs along the path/trail with information concerning wetland values and biodiversity?	YES +1	NO
	TOTAL SCORE /6	

Condition should be noted as FINE; VANDALISED; BROKEN; UNUSABLE

**FACILITIES AND AMENITIES**	**Condition**

Are there toilets?	YES +1	NO	
IF YES is there a disabled toilet?	YES +1	NO	
Are there benches?	YES +1	NO	
Are there barbeques?	YES +1	NO	
Is there a fireplace?	YES +1	NO	
Are there water refill stations?	YES +1	NO	
Are there picnic tables?	YES +1	NO	
Are there rubbish bins?	YES +1	NO	
IF YES are the rubbish bins overflowing?	YES -1	NO	*Leave blank*
Is camping permitted?	YES +1	NO	*Leave blank*
Are there showers?	YES +1	NO	
Is there a boat ramp?	YES +1	NO	
Is there a bird hide?	YES +1	NO	

Are there viewing platforms?	YES +1	NO	
Is there a playground?	YES +1	NO	
	IF CONDITION IS UNUSABLE -1<br>IF CONDITION IS VANDALISED/BROKEN -0.5<br>TOTAL SCORE /14		

**FIRST NATIONS SITE VALUES AND ELEMENTS**		
Are elements within the site significant to First Nations people indicated by signs?	YES +1	NO
Is information specific to these elements available onsite?	YES +1	NO
Is access to those elements restricted by fences or other barriers?	YES +1	NO
Is there evidence of vandalism to those elements?	YES -1	NO
	TOTAL SCORE /3	

**MAINTENCE AND MANAGEMENT**		
Is there evidence that the site is being managed? (e.g., mown grass)	YES +1	NO

	TOTAL SCORE /1

NATURAL VALUES		
Is the site human-made?	YES	NO
Is there signage regarding human-induced threats to the natural values of the site?	YES +1	NO
Are there fences or barriers preventing/restricting access to areas within the site to protect natural values?	YES +1	NO
IF YES is there signage indicating why the fences/barriers are in place?	YES +1	NO
Is there any evidence that restoration activities been undertaken at the site?	YES +1	NO
IF YES Is there signage indicating that this restoration has occurred?	YES +1	NO
Is there signage indicating floral values of the site?	YES +1	NO
Is there signage indicating faunal values of the site?	YES +1	NO

	TOTAL SCORE /7

Table S7. Results of Wetland Wander Tool (WWT) assessments undertaken across 21 wetlands in Tasmania during the summer months (January) of 2024.

**Domain**	**Findings**
Signage	The results of the domain Signage shown in Table 3 (in the main manuscript) highlight that the wetlands assessed are highly variable in terms of the signs they have, with relatively even scores amongst sites with low, fair, moderate, and high. As indicated in Table 3 all sites had some form of signage, therefore lower and higher range scores were determined by the content and condition of the signs rather than by their presence (or absence). Most sites had signs indicating the location of a wetland at its boundary. Sites which scored moderate to high mostly had signage relating to natural values. Sites lost points if signs were damaged, illegible or obstructed.
Online Presence	The results of the domain Online Presence shown in Table 3 highlights that the wetlands studied are mostly uniform in terms of their internet visibility as assessed by the tool. Wetlands scored in this domain tended to be in the low to none range (71%, n=15). Points were lost most frequently in this domain because if a webpage did showcase a wetland, it was not visible through keyword phrase searches (e.g. “places to see near...”). This meant the search results of the general area a wetland was in (e.g. Coles Bay or Freycinet National Park for Moulting Lagoon) did not reveal any webpage mentioning the wetland site as a place to visit. Keyword phrases were determined from *Google Ads Keyword Planner*, a website which outlines the most frequently searched phrases in relation to a given topic.
Education and Outreach	The results of the domain Education and Outreach shown in Table 3 highlight that the wetlands assessed in this study are mostly uniform in terms of their promotion of education and outreach activities as assessed by the tool. Most wetlands in this domain scored in the range none (76%, n=16). Therefore only 24% (n=5) of wetlands studied were awarded points

	in this domain. Wetlands which scored points in this domain did so as they offered events, ran tours, had visitor centres, or promoted volunteer work.
Safety and Security	The results of the domain Safety and Security shown in Table 3 highlight that most of the wetlands studied provide visitors a safe and secure environment as defined by the tool criteria. Almost every wetland studied scored either in the moderate (43%, n=9) or high (48%, n=10) range. Wetlands lost points if they did not mention hazards (e.g. snakes) or were enclosed by vegetation increasing risk factors (e.g. fire and anti-social behaviour).
Access	The results of the domain Access shown in Table 3 highlight that most wetlands studied provide access to sites based on the tools criteria. Almost all wetlands studied in this domain scored in the moderate range (90%, n=19). Wetlands which did not score in this range (7%, n=3) did so because entry to the wetland was either hindered by natural barriers like dense vegetation or a lack of parking. Almost every wetland lost points in this domain due to a lack in the availability of public transport. Those which did not lose points for this were in or close to urban areas.
Aesthetics	The results of the domain Aesthetics shown in Table 3 highlight the variability amongst wetlands assessed, but also that all wetlands assessed scored points. Wetlands in this domain were evenly distributed between the fair (43%, n=9) and moderate-high ranges (38%, n=8). Points were commonly lost in this domain due to a lack of facilities/amenities (e.g. benches) available which would enhance a person's ability to appreciate the aesthetics of the wetland.
Facilities/ Amenities	The results of the domain Facilities/Amenities shown in Table 3 highlight that wetlands included in this study are highly variable in what they offer to visitors in terms of facilities and amenities (e.g. toilets, rubbish bins, viewing platforms, benches, campgrounds). Wetland scores in this domain trended slightly more towards the low range (43%, n=9). There where

	however several wetlands which scored within the fair, moderate, and high ranges (52%, n=11). High scoring wetlands had more facilities and amenities than low scoring wetlands. Although points could be deducted due to the condition of the facilities and amenities no points were deducted from any wetland.
Natural Values	The results of the domain Natural Values shown in Table 3 highlight that most wetlands in this study showed visible efforts to protect or restore the natural values present, or to educate visitors on the faunal and floral values on-site. Wetland scores in this domain were mostly in the fair range (48%, n=10). Results were evenly spread on either side of the fair range, with none-low (29%, n=6) and moderate-high (24%, n=5). Sites frequently lost points as they did not inform visitors of human-induced threats to the area and did not indicate why fences or barriers were in place. Points were awarded to many wetlands for fences/barriers which protected natural values.
Paths/ Trails	The results of the domain Paths/Trails shown in Table 3 highlight that most wetlands in this study did not have paths or trails. The largest portion of wetlands scored in the range none (43%, n=9) and therefore contained no paths. If paths were present, points were lost as there was either no indication of where the path went (e.g. directional signs), no places to sit along the path (e.g. benches), or no signs along the path to indicate natural values.
Accessibility	The results of the domain Accessibility shown in Table 3 highlight that most of the wetlands in this study do not provide access for people with diverse physical and cognitive needs as based on the tools criteria. Wetlands scored mostly in the range none (48%, n=10). Therefore, few wetlands were provisioned with facilities and amenities such as disabled parking and disabled toilets. Few wetlands also provided pathways suitable for wheelchairs, signs for those with vision impairment and signs in

	languages other than English. Assessment criteria for this section was partly developed using *The Australian Standard: Design for access and mobility AS 1428.1 - 2009*
First Nations Values and Elements	The results of the domain First Nations Values and Elements shown in Table 3 highlight that most wetlands in the study gave no acknowledgement of the First Nation values and elements within their boundaries. Wetlands scored mostly in the range none 90% (n=19). Wetlands which were awarded points acknowledged First Nations values and elements through signs, in guides and online.

Table S8. Results for every item tested for inter-rater reliability.

Domain	Overall agreement	Item	Percentage agreement (%)	Weighted/ unweighted	Cohen's kappa (*k*)	Level of agreement
Education and outreach	Near perfect (0.96)	1	100	UW	1	Perfect
		2	100	UW	1	Perfect
		3	100	UW	1	Perfect
		4	95	UW	0.64	Substantial
		5	100	UW	1	Perfect
		6	100	UW	1	Perfect
		7	100	UW	1	Perfect
		8	100	UW	1	Perfect
		9	95	UW	0.83	Near Perfect
		10	100	UW	1	Perfect
Use of site	Fair (0.40)	1	70	W	0.75	Substantial
		2	67	UW	0.33	Fair
		3	79	UW	0.47	Moderate
		4	59	UW	0.13	Slight
		5	83	UW	0.57	Moderate
Signage	Substantial (0.77)	1	82	UW	0.60	Moderate
		2	95	UW	0.83	Near perfect
		3	81	UW	0.61	Substantial
		4	94	UW	0.87	Near Perfect
		5	94	UW	0.77	Substantial
		6	84	UW	0.68	Substantial
		7	94	UW	0.82	Near perfect
		8	89	UW	0.79	Substantial
		9	1	UW	1	Perfect
		10	1	UW	1	Perfect
		11	94	UW	0.77	Substantial
		12	94	UW	0.82	Near Perfect

Access	Substantial (0.78)	1	83	UW	0.61	Substantial
		2	1	UW	1	Perfect
		3	95	UW	0.64	Substantial
		4	70	W	0.66	Substantial
		5	91	UW	0.61	Substantial
		6	83	UW	0.66	Substantial
		7	95	UW	0.64	Substantial
		8	83	UW	0.67	Substantial
		9	91	UW	0.81	Near perfect
		10	91	UW	0.81	Near perfect
		11	1	UW	1	Perfect
		12	1	UW	1	Perfect
Accessibility	Near Perfect (0.84)	1	59	W	0.40	Fair
		2	67	W	0.5	Moderate
		3	90	UW	0.77	Substantial
		4	90	UW	0.62	Substantial
		5	80	UW	0.58	Moderate
		6	95	UW	0.83	Near Perfect
		7	100	UW	1	Perfect
		8	100	UW	1	Perfect
Safety and security	Near perfect (0.82)	1	83	UW	0.67	Substantial
		2	80	UW	N/A	N/A
		3	91	UW	0.74	Substantial
		4	100	UW	1	Perfect
		5	95	UW	0.77	Substantial
		6	91	UW	0.62	Substantial
		7	100	UW	1	Perfect
		8	N/A	W	N/A	N/A
		9	N/A	W	N/A	N/A
Incivilities	Moderate (0.51)	1	65	UW	0.005	Poor
		2	N/A	W	N/A	N/A
		3	87	UW	0.36	Fair

		4	95	UW	0.83	Near perfect
		5	73	UW	0.19	Slight
		6	100	UW	1	Perfect
Aesthetics	Substantial (0.67)	1	91	UW	0.62	Substantial
		2	95	UW	0.77	Substantial
		3	1	UW	1	Perfect
		4	83	UW	0.40	Fair
		5	1	UW	1	Perfect
		6	0.8	UW	0.53	Moderate
		7	87	UW	0.64	Substantial
		8	77	UW	0.46	Moderate
		9	87	UW	0.64	Substantial
		10	67	UW	0.15	Slight
		11	83	UW	0.57	Moderate
		12	77	UW	0.50	Moderate
		13	77	UW	0.54	Moderate
		14	83	UW	0.52	Moderate
		15	91	UW	0.67	Substantial
		16	67	UW	0.40	Fair
Paths/trails	Substantial (0.60)	1	91	UW	0.74	Substantial
		2	100	UW	1	Perfect
		3	62	UW	0.16	Slight
		4	67	UW	0.33	Fair
		5	90	UW	0.62	Substantial
		6	N/A	N/A	N/A	N/A
		7	82	UW	0.61	Substantial
		8	82	UW	0.65	Substantial
		9	82	UW	0.65	Substantial
		10	90	UW	0.74	Substantial
Facilities/ amenities	Near perfect (0.86)	1	83	UW	0.68	Substantial
		2	100	UW	1	Perfect
		3	87	UW	0.74	Substantial

		4	91	UW	0.82	Near perfect
		5	87	UW	0.60	Moderate
		6	95	UW	0.90	Near perfect
		7	91	UW	0.81	Near perfect
		8	91	UW	0.81	Near perfect
		9	100	UW	1	Perfect
		10	100	UW	1	Perfect
		11	95	UW	0.88	Near perfect
		12	95	UW	0.90	Near perfect
		13	100	UW	1	Perfect
		14	90	UW	0.74	Substantial
		15	95	UW	0.83	Near perfect
		16	100	UW	1	Perfect
Maintenance/ management	Perfect (1)	1	100	UW	1	perfect
First Nations	N/A	1<br>2<br>3<br>4	N/A	N/A	N/A	N/A
Natural features	Substantial (0.62)	1	80	UW	0.23	Fair
		2	83	UW	0.25	Fair
		3	86	UW	0.72	Substantial
		4	86	UW	0.73	Substantial
		5	83	UW	0.56	Moderate
		6	95	UW	0.90	Near perfect
		7	86	UW	0.58	Moderate
		8	86	UW	0.64	Substantial
