# Supplementary material for: Assessing human–nature connection: A systematic review and a new Wetland Wanderer Tool for auditing nature connection in wetland environments
Source: Ambio. 2026 Feb 20;55(8):1933–53. doi: 10.1007/s13280-025-02335-1 (PMC13319621; doi:10.1007/s13280-025-02335-1)

Wetland Wanderer Tool

Version 4 2025

Kate Pratt & Vishnu Prahalad
University of Tasmania
Vishnu.Prahalad@utas.edu.au

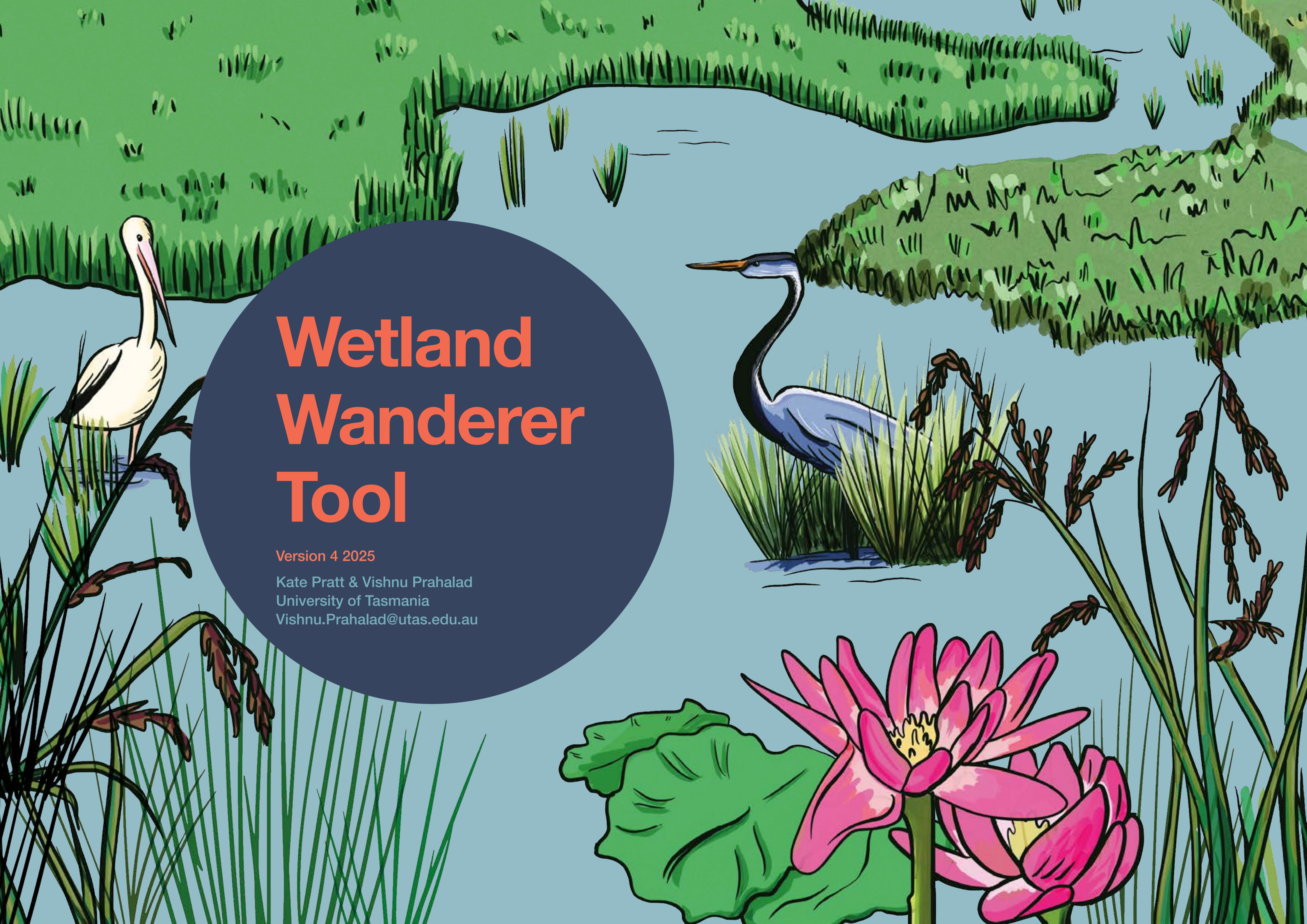

Site assessors contact details:

Assessor name: ....

Email: .... Phone: ....

Name of Site: ....

Type of wetland: ....

Desktop analysis

Location

Address: ....
Local Government Area: ....
Management agency: ....
Distance (km) to nearest Urban Centre: ....

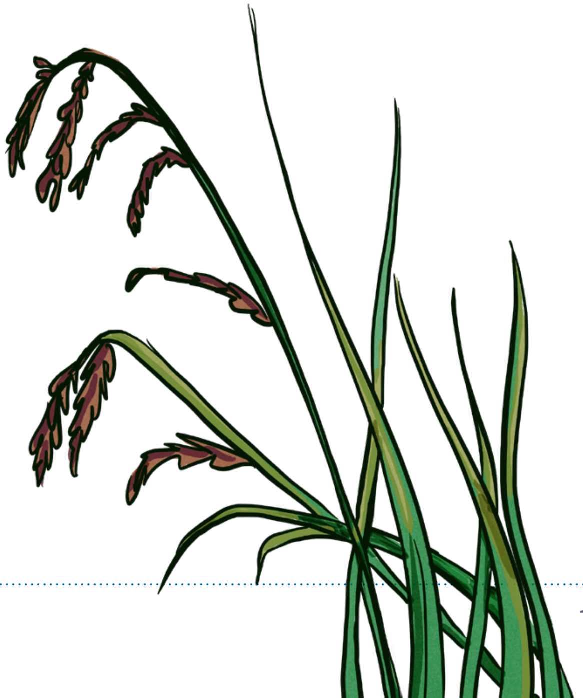

Online Presence

Name of Site		
Type of wetland (e.g. lagoon; lake; saltmarsh)		
Can information concerning recreational opportunities at the site be located via a search engine?	YES	NO
Protection status (e.g. Ramsar Convention; IUCN; National Reserve System)		
Cultural Heritage status (e.g. World Heritage)		
First Nations Heritage status (If not available use AIATSIS map)		
Are there any volunteer opportunities advertised online? (e.g. Parks and Wildlife Service; Government websites; NRM)	YES	NO
NOTES		

Field analysis

Date: .... / .... / .... Time of visit: .... to ....

Weather (e.g. sunny / cloudy / raining / windy): ....

Temperature: ....

Education and Outreach		
Are there volunteering opportunities advertised onsite	YES	NO
IF YES how are these opportunities advertised and what are they?		
Are there any events advertised?	YES	NO
IF YES how are these events advertised and what are they?		
Are tours of the site available?	YES	NO
IF YES are the tours focused on wetland values and biodiversity?	YES	NO
Is there a Visitor Centre attached to the site?	YES	NO
IF YES does the centre have a focus on information specific to wetlands?	YES	NO

Site Popularity	
How many people are onsite?	0 <10 10-30 30-50 50+
What activities are people undertaking?	Bird watching, Picnic/BBQ, Trail walking, Bike riding, Photography, Kayaking, Camping, Boating, Fishing, Volunteering, Hunting, Working
If people are undertaking activities not listed above, please indicate what they are here	
What activities can you see indirect evidence of (e.g. signs; campground)	Bird watching, Picnic/BBQ, Trail walking, Bike riding, Photography, Kayaking, Camping, Boating, Fishing, Volunteering, Hunting, Working
If there is evidence of activities not listed above, please indicate what they are here	

Condition should be noted as FINE; OBSTRUCTED; DAMAGED; ILLEGIBLE

**Signage**			**Condition**
Is there signage at the site's boundary indicating its presence?	YES	NO	
Is there signage within the site? (IF NO please move to ACCESS)	YES	NO	*Leave blank*
Does signage contain educative information about natural values and biodiversity?	YES	NO	
IF YES is the information specific to wetland values and biodiversity?	YES	NO	*Leave blank*
Does signage contain information outlining what activities are prohibited onsite?	YES	NO	
IF YES what activities are prohibited?			
Is there signage which indicates who the tradition owners of the site are?	YES	NO	
Is there signage with a map of the site?	YES	NO	
Is there signage with information regarding who manages the site?	YES	NO	
Is there signage with information regarding volunteer opportunities?	YES	NO	
Is there signage encouraging Citizen Science? (e.g. iNaturalist)	YES	NO	

**Signage**		**Condition**
If there are other forms of signage, please indicate what information they relay		
NOTES		

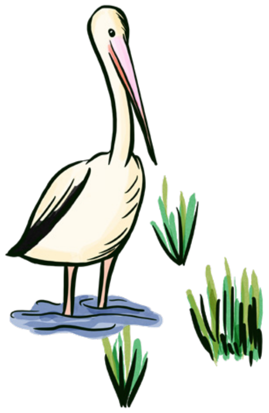

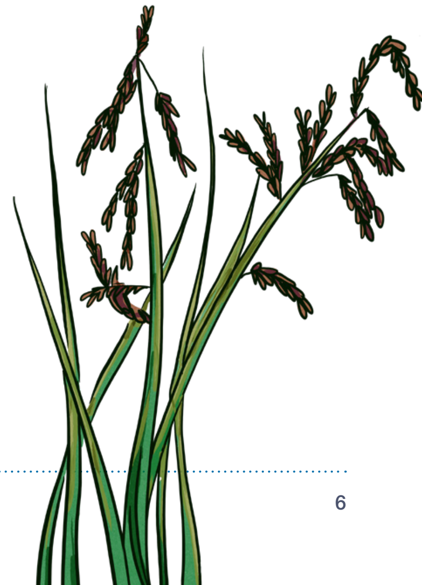

Access		
Is there any indication of the site's existence from the nearest main road?	YES PARTIALLY NO	
Can you enter the site?	YES	NO
IF NO is there a designated point along the road to view the site?	YES	NO
If you can enter the site how many entrance points are there?	1 2 3–5 5+	
Is car parking available?	YES PARTIALLY NO	
IF YES is parking specifically for visitors to the site?	YES	NO
Is parking less than 1 km from the site?	YES	NO
Is the road to the site sealed?	YES PARTIALLY NO	
IF NO is the road to the site FWD access only?	YES	NO
Is public transport available <1 km from the site?	YES	NO
NOTES		

Accessibility		
Is the path/trail smooth, slip resistant and free from uneven surfaces?	YES PARTIALLY NO	
IF YES/PARTIALLY does the path/trail have a width of 1 m or greater at all times?	YES PARTIALLY NO	
IF PARTIALLY for the above two questions are these sections located at the entrance point and do they enable access to facilities/amenities	YES	NO
IF YES what facilities/amenities		
Is there disability parking available?	YES	NO
Is the site accessible for people with vision impairment? (e.g. braille on signs)	YES	NO
Does the site have signage in languages other than English?	YES	NO
IF YES which languages?		
NOTES		

Safety and Security		
Are there any hazards which are not indicated by signs? (i.e. crocodiles; exposed cliff faces)	YES	NO
Is there mobile phone reception?	YES PARTIALLY NO	
Is anyone onsite exhibiting anti-social behaviour? (e.g. intoxication; loitering)	YES	NO
Do you feel safe?	YES PARTIALLY NO	
*If there are no trails within the site, move to **INCIVILITIES***		
Are trails within the site enclosed by vegetation? (e.g. can you see houses, the road, and/or exits?)	YES PARTIALLY NO	
Are the trail edges clearly defined? (e.g. is the vegetation along the edges overgrown and/or containing rubbish?)	YES PARTIALLY NO	

Incivilities		
Is there litter present?	YES	NO
IF YES please indicate the amount	LOW MODERATE HIGH	
Is there evidence of vandalism? (e.g. human caused damage)	YES	NO
Is there any evidence of alcohol or drug use?	YES	NO
Is there any sex paraphernalia present? (e.g. condoms)	YES	NO

Aesthetics		
Can you see the wetland from the nearest road?	YES	NO
Is there a designated place to view the wetland from the road?	YES	NO
IF YES is the view impeded? (e.g. buildings; vegetation)	YES	NO
Is there a place to sit and view the wetland?	YES	NO
If you cannot access the site please move to PATHS/TRAILS		
Can you see the wetland within the site?	YES	NO
Are the views impeded by anything? (e.g. buildings; vegetation)	YES	NO
Is there a place to sit and view the wetland?	YES	NO
Are there any pleasant smells? (e.g. flowers)	YES	NO
Are there any unpleasant smells? (e.g. rubbish)	YES	NO
Are there any pleasant noises? (e.g. birds; water)	YES	NO
Are there any unpleasant noises? (e.g. cars; machinery)	YES	NO
NOTES		

Paths / Trails		
Are there trails/paths around/within the site? (IF NO move to FACILITIES / AMENITIES)	YES PARTIALLY NO	
Is there a sign indicating where paths/ trails lead?	YES	NO
Is the path/trail material consistent throughout?	YES	NO
What is the path/trail material? (circle all that apply)	Dirt<br>Gravel<br>Sand	Board walk<br>Concrete<br>Other:<br>...........................
Are there stairs along the path/trail	YES	NO
IF YES how many?		
Does the trail have uphill sections?	YES	NO
Are there places to sit along the path/ trail?	YES	NO
IF YES are these places located within 50m of each other?	YES	NO
Are there signs along the path/trail with information concerning wetland values and biodiversity?	YES	NO
NOTES		

Condition should be noted as FINE; VANDALISED; BROKEN; UNUSABLE

Facilities / Amenities			Condition
Are there toilets?	YES	NO	
IF YES is there a disabled toilet?	YES	NO	
Are there benches?	YES	NO	
Are there barbeques?	YES	NO	
Is there a fireplace?	YES	NO	
Are there water refill stations?	YES	NO	
Are there picnic tables?	YES	NO	
Are there rubbish bins?	YES	NO	
IF YES are the rubbish bins overflowing?	YES	NO	*Leave blank*
Is camping permitted?	YES	NO	*Leave blank*
Are there showers?	YES	NO	
Is there a boat ramp?	YES	NO	
Is there a bird hide?	YES	NO	
Are there viewing platforms?	YES	NO	
IF YES are there places to sit on viewing platforms?	YES	NO	
Is there a playground?	YES	NO	

NOTES

First Nations Site Values / Elements

Are elements within the site significant to First Nations people indicated by signs? *(IF NO move to NATURAL FEATURES)*	YES PARTIALLY NO	
Are the locations of these elements disclosed?	YES	NO
Is access to those elements restricted by fences or other barriers?	YES	NO
Is there evidence of vandalism to those elements?	YES	NO

NOTES

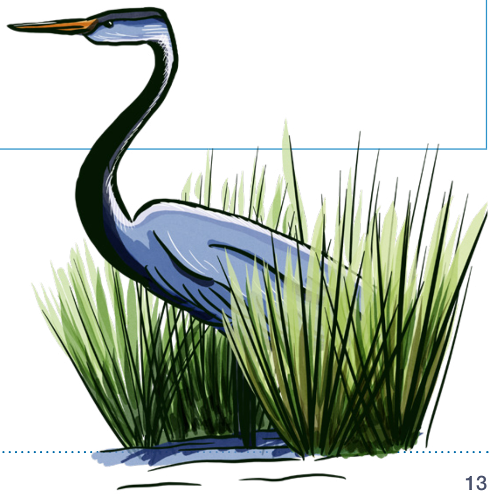

The development process of the Wetland Wanderer Tool is outlined in a research paper published in *Ambio*, titled 'Assessing human-nature connection: A systematic review and a new Wetland Wanderer Tool for auditing nature connection in wetland environments'

Natural Features

Is the site human-made?	YES PARTIALLY NO	
Is there signage regarding human-induced threats to the natural values of the site?	YES	NO
Are there fences or barriers preventing / restricting access to areas within the site to protect natural values?	YES	NO
IF YES is there signage indicating why the fences/barriers are in place?	YES	NO
Is there any evidence that restoration activities been undertaken at the site?	YES	NO
IF YES Is there signage indicating that this restoration has occurred?	YES	NO
Is there signage indicating floral values of the site?	YES	NO
Is there signage indicating faunal values of the site?	YES	NO

NOTES

This tool was developed by Kate Pratt & Vishnu Prahalad,
School of Geography, Planning & Spatial Sciences,
University of Tasmania, Hobart, Australia

Correspondence: Vishnu.Prahalad@utas.edu.au

For more information about the development and application of this this Wetland Wanderer Tool, see the associated journal paper.

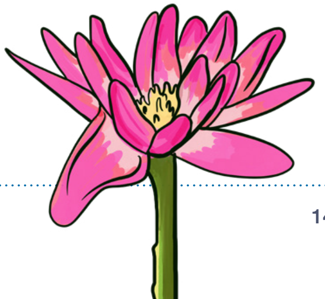

Supplement: Supplementary file 2 — Supplementary file2 (PDF 3292 KB) [file 13280_2025_2335_MOESM2_ESM.pdf]
