# Supplementary material for: Assessing human–nature connection: A systematic review and a new Wetland Wanderer Tool for auditing nature connection in wetland environments
Source: Ambio. 2026 Feb 20;55(8):1933–53. doi: 10.1007/s13280-025-02335-1 (PMC13319621; doi:10.1007/s13280-025-02335-1)
Supplement: Supplementary file 4 — Supplementary file4 (PDF 220 KB) [file 13280_2025_2335_MOESM4_ESM.pdf]

Appendix Material 1 (Appendix Tables A1.1 - A1.2) for

Assessing human-nature connection: A systematic review and a new Wetland Wanderer Tool for auditing nature connection in wetland environments

Kate Pratt [AB]*, Vishnu Prahalad* [A]

[A] School of Geography, Planning and Spatial Science, University of Tasmania, Hobart 7001, Tasmania, Australia

[B] Corresponding author. Email: kate.pratt@utas.edu.au

July 2024

Appendix 1 Information regarding tools included in review

Table A1.1: The name, common abbreviated form and reference for all 14 tools included in our systematic literature review.

**Tool Name**	**Acronym**	**Reference**
BlueHealth environmental assessment tool	BEAT	Mishra, H.S., S. Bell, P. Vassiljev, F. Kuhlmann, G. Niin, and J. Grellier. 2020. The development of a tool for assessing the environmental qualities of urban blue spaces. *Urban Forestry and Urban Greening* 49. https://doi.org/10.1016/j.ufug.2019.126575
Bedimo Rung Assessment Tool – Direct Observation	BRAT-DO	Bedimo-Rung, A.L., J. Gustat, B.J. Tompkins, J. Rice, and J. Thomson. 2006. Development of a Direct Observation Instrument to Measure Environmental Characteristics of Parks for Physical Activity. *Journal of Physical Activity and Health* 3: 176-189. https://doi.org/10.1123/jpah.3.s1.s176
Community Park Audit Tool	CPAT	Kaczynski, A.T., S.A. Wilhelm Stanis, and G.M. Besenyi. 2012. Development and Testing of a Community Stakeholder Park Audit Tool. *American Journal of Preventive Medicine* 42:242-249. https://doi.org/10.1016/j.amepre.2011.10.018
Environmental Assessment of Public Recreation Spaces tool	EARPS	Saelens, B.E., L.D. Frank, C. Auffrey, R.C. Whitaker, H.L. BurdeWe, and N. Colabianchi, N. 2006. Measuring Physical Environments of Parks and Playgrounds: EAPRS Instrument Development and Inter-Rater Reliability. *Journal of Physical Activity and Health* 3: S190-S207. https://doi.org/10.1123/jpah.3.s1.s190
Mexican Public Open Spaces tool	MexPOS	Medina, C., A. Hernandez, M.E. Hermosillo-Gallardo, C.I.G. Gamez, E. Resendiz, M. Morales, C. Nieto, M. Moreno, and S. Barquera. 2022. Development and Validation of the Mexican Public Open Spaces Tool (MexPOS). *International Journal of Environmental Research and Public Health* 19. https://doi.org/10.3390/ijerph19138198
Natural Environment Scoring Tool	NEST	Gidlow, C., E. van Kempen, G. Smith, M. Triguero-Mas, H. Kruize, R. Gražulevičienė, R., Ellis, N., G. Hurst, et al. 2018. Development of the natural environment scoring tool (NEST).

		Urban Forestry and Urban Greening 29:322-333. https://doi.org/10.1016/j.ufug.2017.12.007
Physical Activity Resource Assessment instrument	PARA	Lee, R.E., K.M. Booth, J.Y. Reese-Smith, G. Regan, G., and H.H. Howard. 2005. The Physical Activity Resource Assessment (PARA) instrument: Evaluating features, amenities and incivilities of physical activity resources in urban neighborhoods. *International Journal of Behavioral Nutrition and Physical Activity* 2. https://doi.org/10.1186/1479-5868-2-13
N/A	PARCS	Perry, M.A., H. Devan, H. Fitzgerald, K. Han, L.T. Liu, and J. Rouse. 2018. Accessibility and usability of parks and playgrounds. *Disability and Health Journal* 11: 221-229. https://doi.org/10.1016/j.dhjo.2017.08.011
Parks activity and recreation among kids tool	PARK	Bird, M. E., Datta, G. D., van Hulst, A., Kestens, Y., & Barnett, T. A. (2015). A reliability assessment of a direct-observation park evaluation tool: the Parks, activity and recreation among kids (PARK) tool. BMC Public Health 15(1):906. https://doi.org/10.1186/s12889-015-2209-0
The Path Environment Audit Tool	PEAT	Troped, P.J., E.K. Cromley, M.S. Fragala, S.J. Melly, H.H. Hasbrouck, S.L. Gortmaker, and R.C. Brownson, R. C. 2006. Development and Reliability and Validity Testing of an Audit Tool for Trail/Path Characteristics: The Path Environment Audit Tool (PEAT). *Journal of Physical Activity and Health* 3: 158-175. https://doi.org/10.1123/jpah.3.s1.s158
Public Open Space Tool	POST	Hooper, P. 2012. Public Open Space Tool. The University of Western Australia. https://www.postool.com.au
Systematic Audit of Green-space Environments	SAGE	Byrne, J., J. Wolch, J. Swift, and C. 2005. SAGE (Systematic Audit of Green-space Environments): Audit Form and Instructions. University of Southern California Center for Sustainable Cities, Los Angeles, California.
The senior park environment assessment in Korea	SPEAK	Lee, H.S. 2022. Developing and testing the senior park environment assessment in Korea (SPEAK) audit tool. *Landscape and Urban Planning* 227. https://doi.org/10.1016/j.landurbplan.2022.104545
Woodlands in and around towns	WIAT	Thompson, C.W., and J. Roe. 2010. Protocol to be followed when using the WIAT Questionnaire and Environmental Audit Tools. Edinburgh College of Art, Edinburgh.

Table A1.2 Themes (or, domains) developed from the 14 tools included in this review. Description provides a brief note of what was included in each theme. ✓ indicates whether a tool had criteria relating to the theme. See Table A1.1 for further details on the tools abbreviated here and their associated references.

		TOOLS													
		BEAT	BRAT-DO	CPAT	EAPRS	MexPOS	NEST	PARA	PARCS	PARK	PEAT	POST	SAGE	SPEAK	WIAT
THEMES	DESCRIPTION														
Site context	Site's location in relation to surrounding area.	✓		✓		✓									
Use of site	Who is using the site and for what purpose?	✓	✓				✓			✓		✓			✓
Signage	Multiple criteria specific to each tools aim.	✓	✓	✓	✓	✓		✓		✓	✓		✓		✓
Access	How can you enter the site? How can you get aroun	✓	✓	✓	✓	✓	✓	✓	✓	✓	✓		✓	✓	✓

	d the site?														
Accessibility	Is anyone excluded from the site due to physical disability?	✓			✓				✓		✓			✓	✓
Safety/ Security	Personal safety.	✓		✓	✓	✓		✓	✓	✓	✓	✓	✓	✓	
Incivilities	Evidence of anti-social behaviour.	✓	✓	✓	✓	✓	✓	✓		✓	✓	✓	✓	✓	✓
Visual aesthetics	Visual quality of the site.	✓	✓	✓	✓		✓	✓	✓	✓	✓			✓	✓
Non-visual aesthetics	Sensory appeal of site other than visual.	✓	✓			✓					✓				✓
Paths / Trails	Presence and condition of paths and trails.	✓	✓	✓	✓	✓	✓	✓	✓	✓	✓	✓	✓	✓	✓
Facilities/ amenities	Presence and condition of facilities and	✓	✓	✓	✓	✓	✓	✓	✓	✓	✓	✓	✓	✓	✓

	amenities.														
Maintenance/ Management	Cleanliness, condition and upkeep of the site.	✓	✓	✓	✓	✓	✓		✓	✓	✓	✓	✓	✓	✓
Cultural features	Consideration of artistic, historical and cultural features.	✓	✓	✓	✓	✓	✓					✓			
Natural features	Consideration of natural elements.	✓	✓		✓	✓	✓			✓		✓	✓	✓	✓
